# Comparing photoelectrochemical water oxidation, recombination kinetics and charge trapping in the three polymorphs of TiO_2_

**DOI:** 10.1038/s41598-017-03065-5

**Published:** 2017-06-07

**Authors:** Benjamin Moss, Kee Kean Lim, Alessandro Beltram, Savio Moniz, Junwang Tang, Paolo Fornasiero, Piers Barnes, James Durrant, Andreas Kafizas

**Affiliations:** 1 Imperial College London, Department of Chemistry, South Kensington Campus, London, SW7 2AZ UK; 2 Tunku Abdul Rahman University College, Department of Mathematics, Kampus Utama, Jalan Genting Kelang, 53300 Kuala Lumpur Malaysia; 3 Department of Chemical and Pharmaceutical Sciences and ICCOM Trieste Research Unit, University of Trieste, via L. Giorgieri 1, 34127 Trieste, Italy; 4 University College London, Department of Chemistry, Gower Street, London, WC1H 0AJ UK

## Abstract

In this article we present the first comparative study of the transient decay dynamics of photo-generated charges for the three polymorphs of TiO_2_. To our knowledge, this is the first such study of the brookite phase of TiO_2_ over timescales relevant to the kinetics of water splitting. We find that the behavior of brookite, both in the dynamics of relaxation of photo-generated charges and in energetic distribution, is similar to the anatase phase of TiO_2_. Moreover, links between the rate of recombination of charge carriers, their energetic distribution and the mode of transport are made in light of our findings and used to account for the differences in water splitting efficiency observed across the three polymorphs.

## Introduction

Many first row transition metal oxides (TMOs) show great potential for use as photoelectrodes for the generation solar fuels due to their abundance, stability and low toxicity^1–3^. However the performance of water splitting devices based on TMOs still falls short what is required for this technology to be commercialized^4–6^. The reasons for this shortfall are often complex and not always entirely understood. Commonly cited factors include the nature and size of the band gap, charge carrier recombination, band edge alignment with respect to the water oxidation and proton reduction potentials, the Fermi-Level energy, the mobility of free minority carriers as well as space charge and surface effects^7–14^.

One interesting and potentially informative case of this wider discussion is the effect of crystal structure on photoactivity. A prominent example of this is the disparity in photoactivity between the polymorphs of TiO_2_. Three phases of TiO_2_ are stable under ambient conditions. Anatase, a metastable phase is widely considered to be the most active for both photoelectrochemical (PEC) water oxidation and photocatalysis, despite a wider band gap of approximately 3.2 eV. Rutile, the thermodynamic phase, has a narrower band gap (3.0 eV) but often exhibits photoactivities up to an order of magnitude less than anatase^15^. The third stable polymorph, brookite has only recently become synthetically accessible with sufficient purity and quantity to allow its properties to be reproducibly investigated^16^. Because of this, a consensus on the physical and photoelectrochemical properties of brookite has not yet been reached. Early reports suggest the physical and photocatalytic performance of brookite to be similar to those of anatase^16^. To the best of our knowledge, no study has investigated the performance of pure brookite for water oxidation.

The reasons for the disparity in photoactivity between the polymorphs are unclear and mirror the wider debate regarding the factors that enhance or diminish photoactivity^12, 17–21^. However, until recently the effect of the properties of trapped carriers on the slow (ms-s) timescales that are most relevant to water oxidation has remained unexamined. Work by Batzill and co-workers using high quality epitaxial films of varying lengths has shown that a significant difference in carrier diffusion length exists between anatase and rutile, with carriers in rutile diffusing half as far as anatase^11^. The diffusion length obtained for rutile by Batzill and co-workers (1.6 ± 0.6 nm) matches literature values for the hole diffusion length (1–10 nm)^22^. In addition, work within our group using transient absorption spectroscopy (TAS) has suggested that differences in the rate at which anatase and rutile photocatalytically degrade a model system may be caused by holes in rutile being less photocatalytically reactive^23^.

Recently, a technique capable of measuring the density of occupied mid-gap trap states (DOTS), known as transient infrared absorption - energy scanning spectroscopy (TRIRA-ESS), was employed by Weng and co-workers to investigate the polymorphs of TiO_2_. This study revealed an exponential tail of trap states extending out from the valance band edge in anatase^24^. Interestingly, optical transitions from these states into the conduction band correspond to wavelengths between 415 and 540 nm - a region of absorption typically associated with photogenerated holes in anatase^23, 25^. Subsequent work using TRIRA-ESS has shown that this feature is absent in rutile, where only deep traps are observed, but present in brookite. Most recently, this line of investigation has also suggested that differences in the DOTS between the phases may be related to their differing capacities for overall water splitting^26, 27^.

If differences in the DOTS measured by Weng and co-workers influence the efficiency of the overall water splitting reaction, it is likely that they also affect the efficiency of the water oxidation half reaction in isolation. This is because different trap depths should result in different kinetic barriers for the movement of carriers between traps. This hypothesis is consistent with the measurements made by Batzill. Further, differences in carrier diffusion length should also make themselves manifest in the electron/hole recombination dynamics of the phases on the *μ*s - s timescale. As well as inhibiting carrier diffusion lengths, deep trapping may also result in a reduction in the energy required to drive the kinetically demanding oxidation of water. Thus correlations between the performance and recombination dynamics of anatase, brookite and rutile might reasonably be expected.

In this study, mesoporous films of anatase, brookite and rutile are physically characterized to demonstrate chemical and phase purity, and to exclude the influence of extrinsic variables, such as surface area, on any trends in photoactivity that are observed. The incident photon to current (IPCE) spectrum under an applied bias of 1.23 V *vs* RHE is used as a measure of water oxidation photoactivity. Finally, transient absorption spectroscopy (TAS) is employed to compare their recombination kinetics. TAS data is then analyzed to provide information about charge trapping in the polymorphs. To the best of our knowledge, this study constitutes the first report of the water oxidation efficiency of brookite as well as the first TAS study of this polymorph on the timescales relevant to the kinetics of water oxidation.

## Results

**Figure 1:**
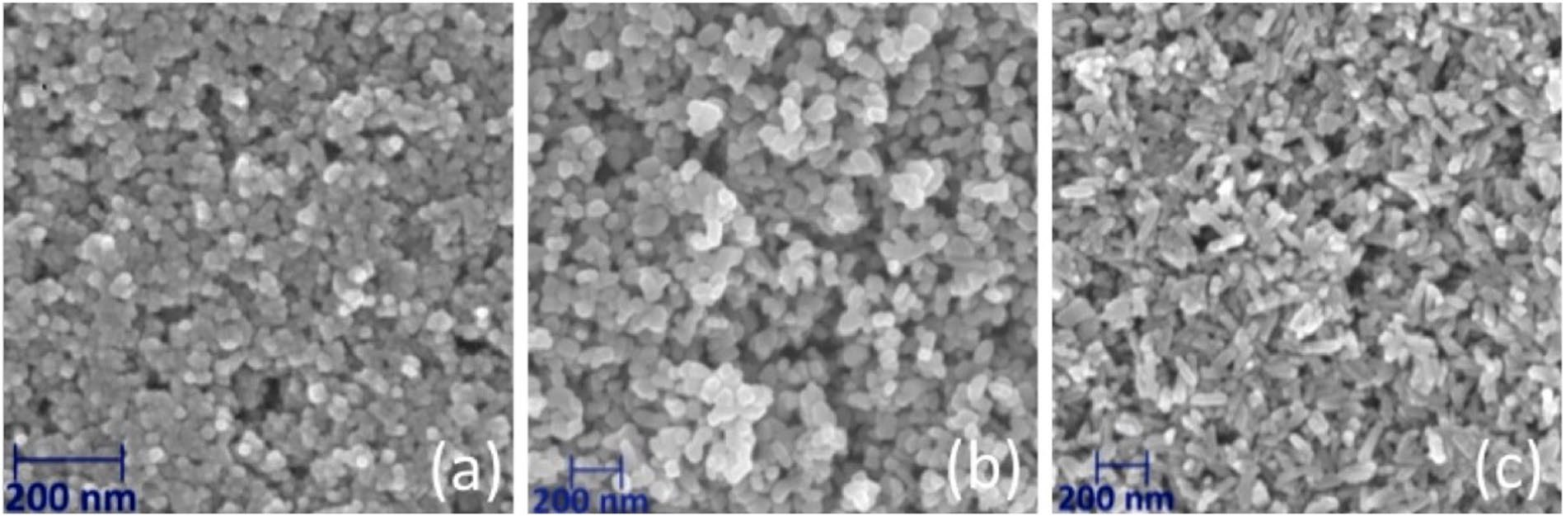
Top-down SEM images of mesoporous (**a**) anatase, (**b**) rutile and (**c**) brookite films on FTO substrates.

SEM images (Fig. 1, Supplementary Fig. S1a) of the anatase, rutile and brookite films show that the particles are evenly distributed to form a mesoporous network. Both SEM and TEM images (Supplementary Fig. S1a and S1b) indicate that particles maintain their integrity and show limited or no Ostwald ripening (necking). The results of PXRD (Supplementary Fig. S2) show a high quality fit to model data in all cases, indicating phase purity. This result is corroborated by the Raman spectra of the films (Supplementary Fig. S3), which compare favorably with standard spectra from the CNISM database. In particular, the absence of shouldering of the 502 *cm* ^−1^ by a second peak at 515 *cm* ^−1^ indicates that brookite is free from contamination by anatase: a commonly reported hurdle in the synthesis of pure brookite^16^.

The results of the physical characterization, summarized in Table 1 below, indicate that the films were highly pure. Binding energies characteristic of Ti, O, and C (presumably arising from the organic binder used to make the colloidal paste and other adventitious carbon residues) were observed in XPS (Supplementary Fig. S4). The carbon signal was strongly diminished with sputtering, indicating chemical purity in the bulk of the films. The optical band gap of all films (Supplementary Fig. S5) also compares favorably to literature values for similar mesopourous films^23, 28^. Although particle shape and size varied between the polymorphs, the specific surface area determined through BET analysis (Supplementary Fig. S6) was commensurate in all phases - with anatase presenting around twice the surface area of brookite or rutile. Such a two-fold difference in surface is unlikely to induce an order of magnitude in efficiency^29^. UV-visible spectroscopy shows the absorptance of anatase and brookite films to be similar over a wide range of wavelengths. Rutile exhibits a raised absorptance due to its narrower bandgap despite and is also more scattering (Supplementary Fig. S7). In order to ensure that light is absorbed evenly throughout the samples, the mesoporous films were fabricated such that the penetration depth of photons with energy greater than the band gap was always slightly less than the film thickness.

**Table 1:** Summary of the results of physical characterization of mesoporous anatase, brookite and rutile.

	Size (nm) and shape	Film thickness (*μ*m)	XPS composition	BET surface area (*m* ^2^ *g* ^−1^)	BJH pore diameter (nm)	Optical band gap (eV)
Anatase	15 spherical	4.5	Ti, O, C (surface)	110	16	3.25
Rutile	58 × 30 oblate	0.9	Ti, O, C (surface)	52	28	3.14
Brookite	123 × 48 rod	0.5	Ti, O, C (surface)	43	62	3.38

**Figure 2:**
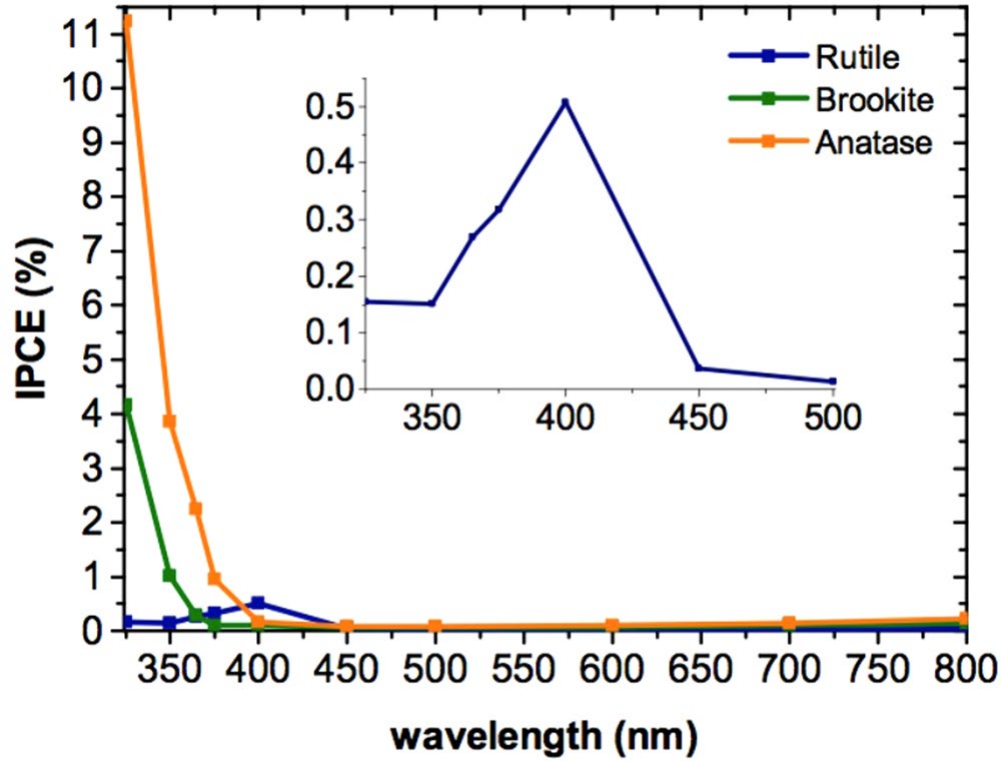
IPCE spectra of mesoporous anatase, brookite and rutile at 1.23 V vs RHE measured at pH 13.6 under front irradiation (semiconductor-electrolyte interface).

A clear trend in the water splitting efficiency of the polymorphs was observed (Fig. 2). The maximum IPCE efficiency of anatase (11.5%) was more than twice that of brookite (4.3%) and 23 times that of rutile (0.5%). The shape of the IPCE spectra is also significant, as the onset of water splitting is earliest in rutile, reflecting its narrower bandgap/earlier onset of absorption. A later onset in brookite suggests a wider bandgap than anatase.

For the anatase and rutile films studied herein, transient absorption (TA) spectra collected under an inert atmosphere and in the presence of chemical scavengers, exhibit similar features to those seen in previous studies^23^. The inert atmosphere spectrum for both polymorphs (Supplementary Fig. S8) shows two overlapping features: a peak between 450 and 550 nm and a broad absorption centered between 800 and 900 nm. Experiments using scavengers (methanol: hole scavenger, silver nitrate: electron scavenger) relate the feature in the blue to the transient absorption of photogenerated holes (Supplementary Fig. S9) whilst the broad feature in the red/near-IR can be attributed to photogenerated electrons (Supplementary Fig. S10). The TA spectrum of brookite (Supplementary Fig. S8) also shows a peak in the blue part of the spectrum and a broad feature in the red/near-IR. As with anatase and rutile, chemical scavenging agents allowed the broad feature in the red/near-IR to be attributed to photogenerated electrons whilst the peak in the blue was assigned to the absorption of photogenerated holes.

**Figure 3:**
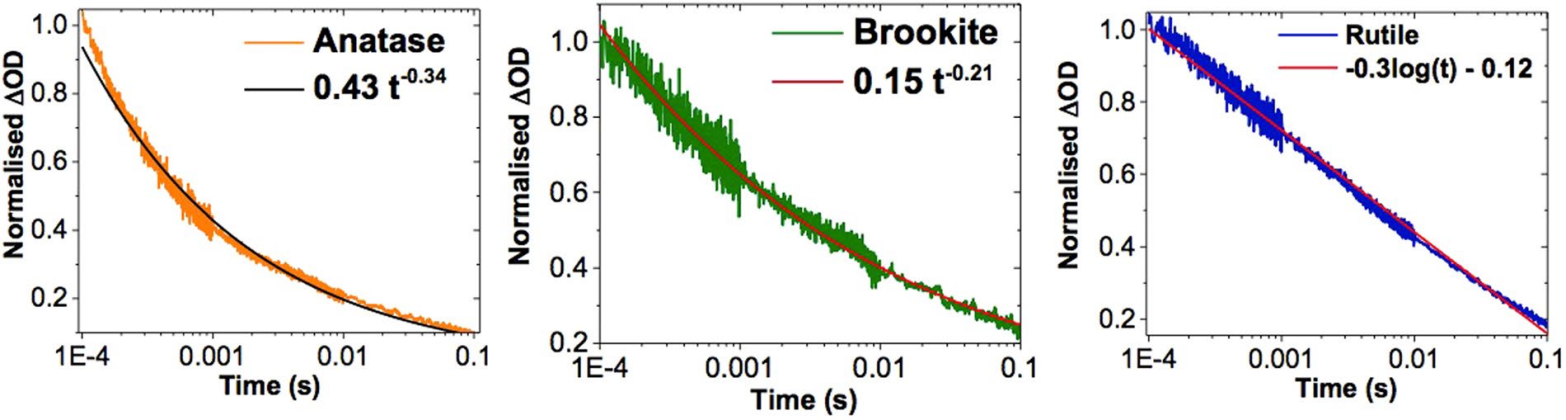
Normalised TA decays of mesoporous anatase at 600 nm (left), brookite at 650 nm (middle) and rutile at 650 nm (right) under an inert atmosphere. These are shown alongside the best fitting equation for each decay. Under an inert atmosphere, the normalized decay kinetics for a given polymorph match at all wavelengths. Consequently, decays corresponding to wavelengths with the highest signal to noise ratio are shown.

The normalized TA decays collected under an inert atmosphere for all polymorphs, show that decays recorded in the electron and hole regions overlap. This shows that for all polymorphs, only electron/hole recombination occurs under an inert atmosphere. The normalized TA dynamics for anatase, brookite and rutile is shown in Fig. 3 below.

TA decays for anatase and brookite exhibit power law dynamics, evidenced by a high quality fit to Eq. 1, which describes a simple power law decay:

1 $$[{h}^{+}](t)=A{t}^{-\alpha }$$

where [h+](t) is the concentration of holes at time, t. A and *α* are fitting constants. The difference in curvature of the TA decays of anatase and brookite is significant. This is reflected in the exponent of the power law (*α*), which is larger for anatase (0.34) than brookite (0.21).

Unlike anatase and brookite, rutile does not exhibit power law recombination kinetics. Rather, a ‘log-linear’ decay is observed. Empirically, we can describe this behaviour with a log decay (Eq. 2), which produces a high quality fit to the data:

2 $$[{h}^{+}](t)=-Alog(t)+c$$

where A and c are fitting constants.

The relationship between trapping and decay dynamics in has been previously understood in TiO_2_ and other defective materials exhibiting power law and ‘stretched exponential’ type recombination dynamics by demonstrating that transient absorption decays can be reproduced using a random walk model^30–32^. To show that this concept is also valid for rutile, which exhibits a log-decay, the recombination dynamics of this polymorph are reproduced using a random walk model in which electrons may both tunnel or thermally walk to reach a stationary hole (Supplementary Fig. S11, see SI for further details).

## Discussion

The XPS, PXRD, Raman spectrum and the optical band gap of the mesoporous films indicate that all three samples are single polymophs with a high degree of chemical purity. Although a two-fold increase in the surface area was observed between anatase/brookite and rutile, this difference is unlikely to be the principle cause of the order of magnitude difference in IPCE observed between rutile and anatase/brookite. This differece may also in part arise from more (or less) reactive surface facets being presented in a given polymorph. A fascinating hypothesis proposed by Can Li and Weng is that these factors are not necessarily mutually exclusive i.e. that changes in the surface, for example surface reconstruction, may profoundly influence the DOTS^26^.

All samples showed long lived TA signals. Interestingly, the half-time (τ) of the TA decays (measured from 100 *μ*s) follows the opposite trend to that seen in the IPCE spectra (i.e. τ_*rutile*_ > τ_*brookite*_ > τ_*anatase*_). The recombination kinetics observed in anatase and brookite are described by a power law, with an exponent (α)  that is less than 1. A power law of this kind is well described in the literature and arises when ideal ‘bimolecular’ electron/hole recombination (a power law decay of order 1) is disrupted by a multiple trapping process, in which a carrier must repeatedly be thermally excited to the band-edge in order to move, and ultimately recombine. This is reflected in the deviation of *α* to values less than 1. Often associated with this kind of decay dynamics are exponential tails of trap states similar to those observed by Weng and co-workers^27^. If, on average, trapped carriers require more energy to thermally excite to the band-edge, the multiple trapping effect will be exacerbated. This in turn will be reflected by a smaller value of *α* ^30–32^. This is the case for the recombination dynamics of brookite compared to anatase, where both samples exhibit bimolecular recombination with an order less than one.

In principle, the exponent of the power law decay can be influenced by multiple trapping of electrons or holes. However, the exponential tail of states falling away from the valance band edge measured by Weng lends credence to the hypothesis that the multiple trapping of holes strongly contributes to the observed dynamics^27^. Quantitatively, this is reflected in differences in the exponent of the power law (*α*) as $\frac{kT}{\alpha }$ is equal to the mean energy of an exponential tail type trap  distribution^30^.

For anatase and brookite, the values of *α* extracted from Fig. 3 correspond to a mean energy of 76 and 122 meV respectively. Given that the valance band of both anatase and brookite is deep compared to the water oxidation potential^16^ even the extreme interpretation of this value (i.e. the case where holes alone contribute to *α* - suggesting more deeply trapped holes in brookite) corresponds to a difference of less than 2 kT at room temperature. From this we suggest that hole trap depth is unlikely to be the cause of a factor of two difference in IPCE between anatase and brookite. This conclusion is consistent with literature results that suggest that brookite often exhibits a similar photocatalytic activity to anatase^16^. However, the increased trapping of one or both carrier species in brookite may explain why photogenerated charges on the *μ*s - s timescale are longer lived compared to anatase. This result suggests that brookite may be a preferable photocatalyst to anatase in applications where charge carrier lifetime is limiting.

**Figure 4:**
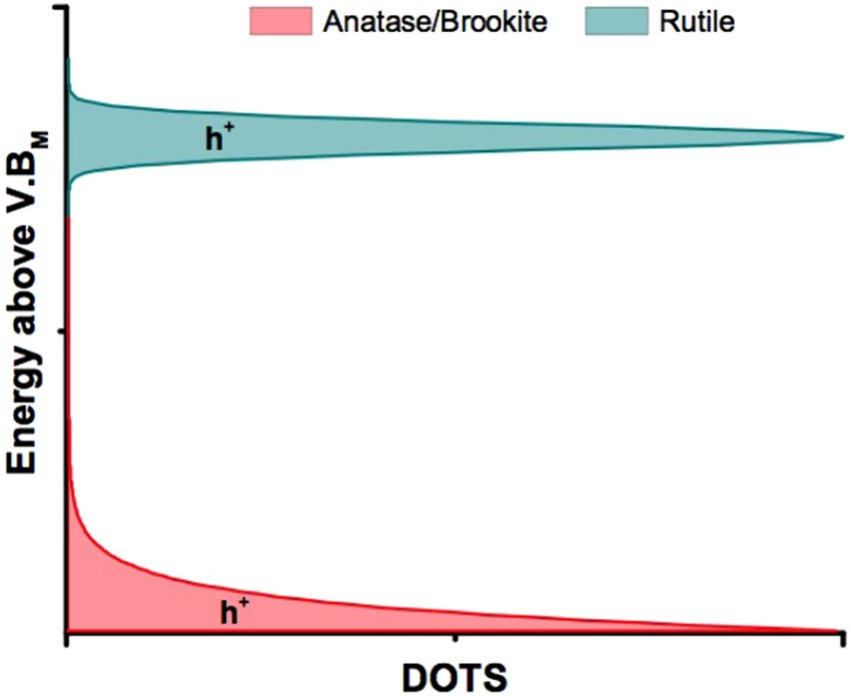
A simplified presentation of possible density of occupied trap states (DOTS) for holes in anatase, brookite and rutile. In anatase and brookite, an exponential tail of shallow traps results in thermal transport, higher mobility and enhanced reactivity. In rutile, a narrow region of deep hole traps causes tunneling mediated recombination, lower mobility and diminished reactivity.

The electron/hole recombination dynamics observed in rutile is unusual as it can be empirically fit by a log decay equation (Eq. 2). These dynamics are not well understood. In previous work we suggested that a log decay might qualitatively be explained by a model in which tunneling contributes significantly to charge transport^23^. In the current work, we note that the log-linear decay dynamics can be quantitatively reproduced to a high degree of accuracy from a random walk model in which an electron may tunnel or thermally walk in order to recombine with an immobile, deeply trapped hole (Supplementary Figs S11 and S12). In the model, a region of log-linear recombination can only be generated in the limiting case where tunneling is the dominant mode of electron transport and recombination mechanism. Although more work is required to demonstrate the versatility of this model, this initial finding supports the hypothesis that log-linear recombination dynamics arise when tunneling dominates over thermal trapping/de-trapping processes. From this, we tentatively assign a log-decay to tunneling mediated recombination arising from the deep trapping of both electrons and holes. This hypothesis is consistent with the smaller diffusion lengths measured by Batzill and co-workers and may also in part explain the extremely poor IPCE of rutile compared to anatase and brookite, by suggesting the presence of holes so deeply trapped that negligible driving force is available to drive water oxidation. This is illustrated in in Fig. 4, which summaries the hole DOTS implied from the analysis of the recombination dynamics of anatase, brookite and rutile. As the recombination dynamics of anatase and rutile has been shown to be independent of morphology (i.e. the same for more crystalline, dense films as for mesoporous films, such as those studied herein) from timescales ranging from the fs-s^23, 33^, we suggest that an analysis of recombination dynamics as a useful indicator of trap mobility and energy in TiO_2_.

In conclusion conceptual links between the rate of recombination of charge carriers, their energetic distribution and mode of transport are used to account for the observed differences in water splitting efficiency in the polymorphs of TiO_2_. Anatase and brookite exhibit dispersive power law recombination dynamics, indicative of shallow charge trapping. Rutile exhibits logarithmic decay kinetics. Using a random walk model, we suggest this to be indicative of deeper charge trapping. In agreement with other studies, we suggest that the presence of deeper charge traps in rutile is a key reason for the poor photoelectrochemical water oxidation performance of this polymorph. On the other hand, relatively efficient water oxidation and longer charge carrier lifetimes are observed for brookite, the least known polymorph of TiO_2_, suggesting that brookite may be a promising choice for photoelectrochemical water oxidation and photocatalytic applications, particularly where lifetime is a limiting factor.

## Methods

Anatase nanoparticles, in the form of a colloidal paste were synthesized according to a previously described method^34^. Rutile nanoparticles were purchased from Aldrich at the highest available purity grade. Brookite nanoparticles were synthesized by hydrothermal treatment at 160 °C for 24 h using commercial titanium (IV) bis(ammonium lactate) dihydroxide aqueous solution (50 wt%, Sigma Aldrich) in the presence of 7 M urea. The white precipitate was collected by centrifugation, washed several times with doubly distilled water and finally dried at 80 °C overnight. This was followed by thermal treatment at 400 °C for 3 hrs in order to remove any organic contaminants coming from the precursor. The particles were then blended into a colloidal paste and doctor- bladed onto an FTO-glass substrate and calcined at 450 °C for one hour using a previously described method^23^.

Powder X-ray diffraction (PXRD) was conducted using a Bruker Lynx-Eye X-ray diffractometer equipped with a monochromated Cu-K*α* (1.5406 Å) X-ray source. Diffractograms were refined using the Le Bail method using GSAS-EXPGUI software against standards from the CDS National Chemical Database^35, 36^. X-ray photoelectron spectroscopy (XPS) was performed using a Thermo Scientific K-*α* instrument. The material bulk was investigated by argon etching. Raman spectroscopy was carried out on a Renishaw Raman microscope and a Leica DMCM microscope. The detection range was from 40–800 *cm* ^−1^. To determine the extent of particle aggregation HRTEM was performed by scraping the mesoporous films into methanol and sonicating them to form a dispersion reflective of the morphology of the particles. The particles were then deposited onto a gold grid and measured using a Jeol 1010 transmission microscope. For SEM, samples were coated with an 100 nm layer of chromium using a Quorum Q150 + S plasma coater. SEM images were then collected in a Leo 15 electron microscope. Ultra-violet visible (UV-Vis) spectroscopy was performed on a Shimadzu UV-vis 2600 spectrophotometer equipped with an integrating sphere, allowing measurement of both absorptance and reflectance. This data was then used to calculate the optical depth, as well as the band gap of the polymorphs using a Tauc plot^37^. Nitrogen gas adsorption isotherms were measured in a Micrometrics Tri-Star gas isothermometer. Results were interpreted using the Brauner-Emmett-Teller (BET) and Barrett-Joyner-Halenda (BJH) methods to estimate the surface area and average pore diameter of the films respectively. The morphology of the mesoporous TiO_2_ films was investigated by scanning electron microscopy (SEM) using a Leo 15 emission microscope after plasma coating with chromium.

IPCE measurements were carried out in a home-made PTFE cell equipped with quartz windows to allow illumination using monochromated light from a 75 W Xe lamp. A three-electrode configuration was used, with the sample as the working electrode, a platinum mesh counter electrode and a silver/silver chloride reference. Measurements were performed at room temperature using a Metrohm Autolab N-series potentiostat in a solution of 1 M sodium hydroxide (pH 13.6). The IPCE measurement was performed by measuring the intensity of the monochromatic light using a power meter at each wavelength, then placing the photoelectrochemical cell at the same distance from the monochromator and measuring the steady state photocurrent at an applied potential of 1.23 V *vs* RHE. The sample was illuminated from the front. The IPCE was then determined using Eq. 3

3 $$IPCE( \% )=\frac{{J}_{SS}}{{I}_{\lambda }}\cdot 100$$

where *J*_*SS*_ (electrons *cm* ^−2^ *s* ^−2^) is the steady state photocurrent density at a given wavelength and *I*_*λ*_ (photons *cm* ^−2^ *s* ^−2^) is the flux of the monochromatic light source.

The charge carrier recombination dynamics of the polymorphs of TiO_2_ on the *μ*s-s timescale was analysed using transient absorption spectroscopy (TAS). The TAS setup used in this work has been described previously^23^. Briefly, light from a 75 W Xe lamp was monochromated to be used as the probe. The probe beam is transmitted through the sample and onto a silicon diode. A laser flash from the third harmonic of a Nd:YAG laser (6 ns temporal pulse width, 355 nm) was used to excite the samples to generate a transient absorption in the probe beam. Unless otherwise specified, the pump intensity was around 1 *mJ* *s* ^−2^. During the experiment, samples were kept in a sealed quartz cuvette under an argon atmosphere unless otherwise specified. To determine the spectral properties of photogenerated electrons and holes in the polymorphs, methanol and a 1 mM solution of silver nitrate were employed as hole and electron scavengers respectively.

### Data availability

Data underlying this article can be accessed on Zenodo at DOI: 10.5281/zenodo.494993 and used under the Creative Commons Attribution licence.

## Electronic Supplementary material

SI for Comparing photoelectrochemical water oxidation, recombination kinetics and charge trapping in the three polymorphs of TiO2
