# Supplementary material for: Comparing photoelectrochemical water oxidation, recombination kinetics and charge trapping in the three polymorphs of TiO2
Source: Sci Rep. 2017 Jun 7;7:2938. doi: 10.1038/s41598-017-03065-5 (PMC5462794; doi:10.1038/s41598-017-03065-5)
Supplement: Supplementary file 1 — SI for Comparing photoelectrochemical water oxidation, recombination kinetics and charge trapping in the three polymorphs of TiO2 [file 41598_2017_3065_MOESM1_ESM.pdf]

Comparing photoelectrochemical water oxidation, recombination kinetics and charge trapping in the three polymorphs of TiO_2

Benjamin Moss, Kee Kean Lim, Alessandro Beltram, Savio Moniz, Junwang Tang, Paolo Fornasiero, Piers Barnes, James Durrant, and Andreas Kafizas

Hydrothermal synthesis of brookite nanorods

Brookite nanoparticles were synthesized by hydrothermal treatment at 160 $°C$ for 24 h using commercial titanium (IV) bis(ammonium lactate) dihydroxide aqueous solution (50 wt%, Sigma Aldrich) in the presence of urea 7 M. The white precipitate was collected by centrifugation, washed several times with doubly distilled water and finally dried at 80 $°C$ overnight. This was followed by thermal treatment at 400 $°C$ for 3 hrs in order to remove any organic contaminants coming from the precursor.

S1a - full size SEM images of anatase, brookite and rutile films

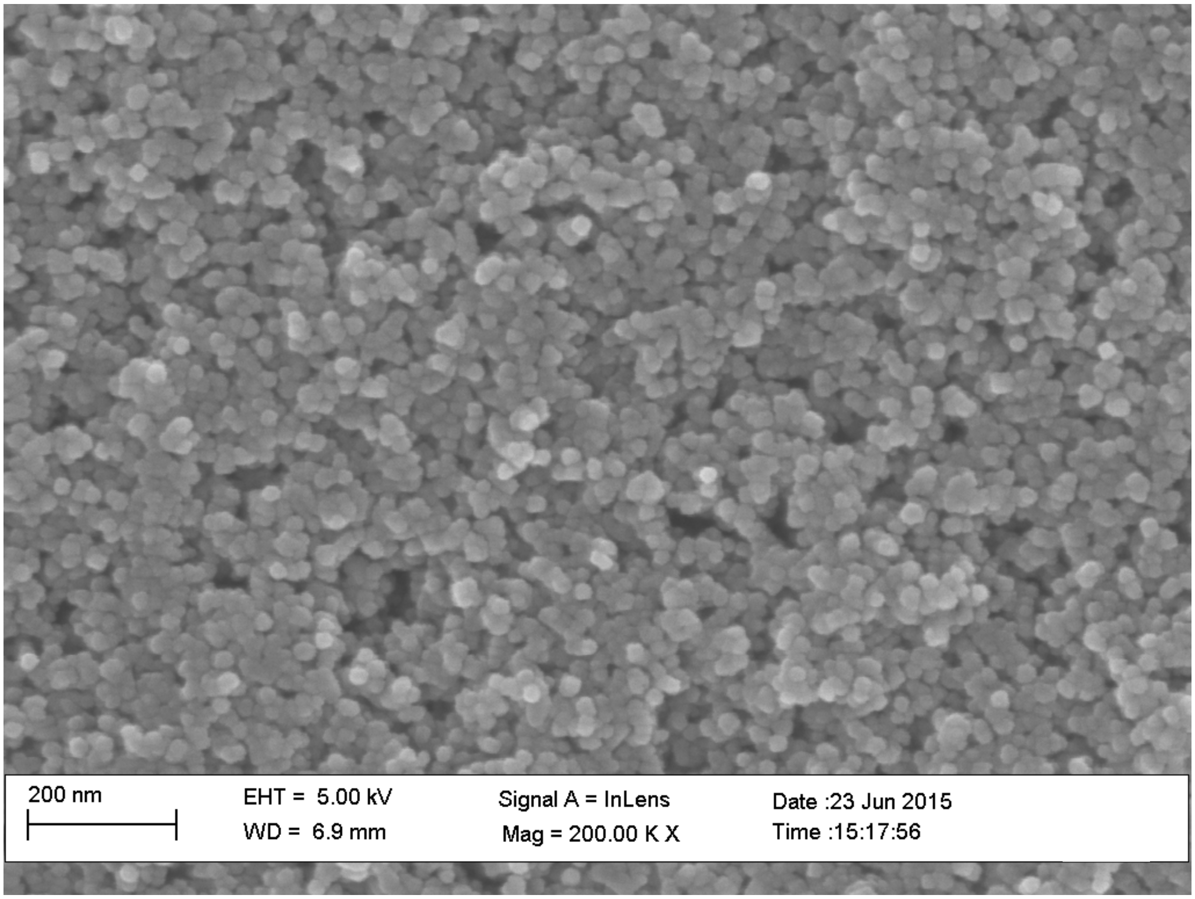

Anatase

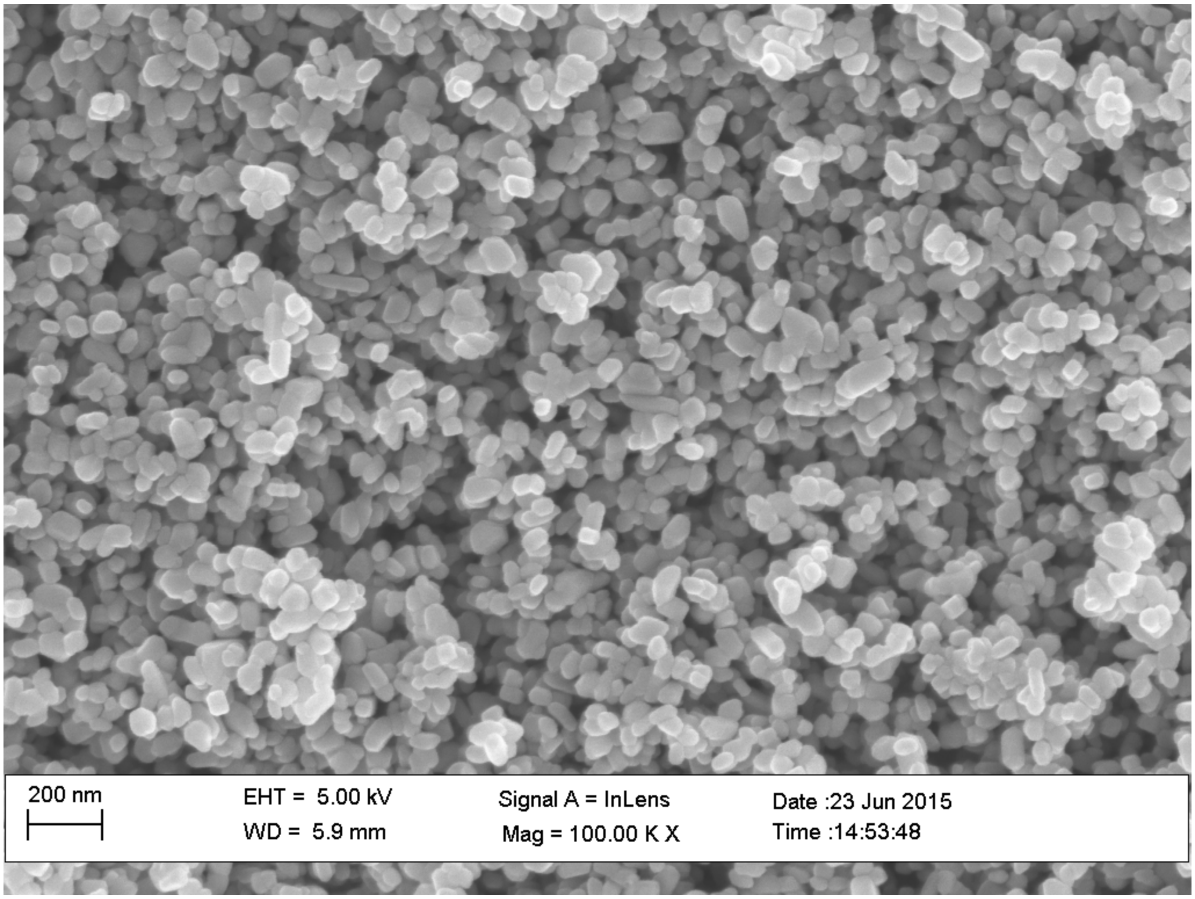
200 nm
EHT = 5.00 kV
WD = 5.9 mm
Signal A = InLens
Mag = 100.00 K X
Date :23 Jun 2015
Time :14:53:48
Rutile

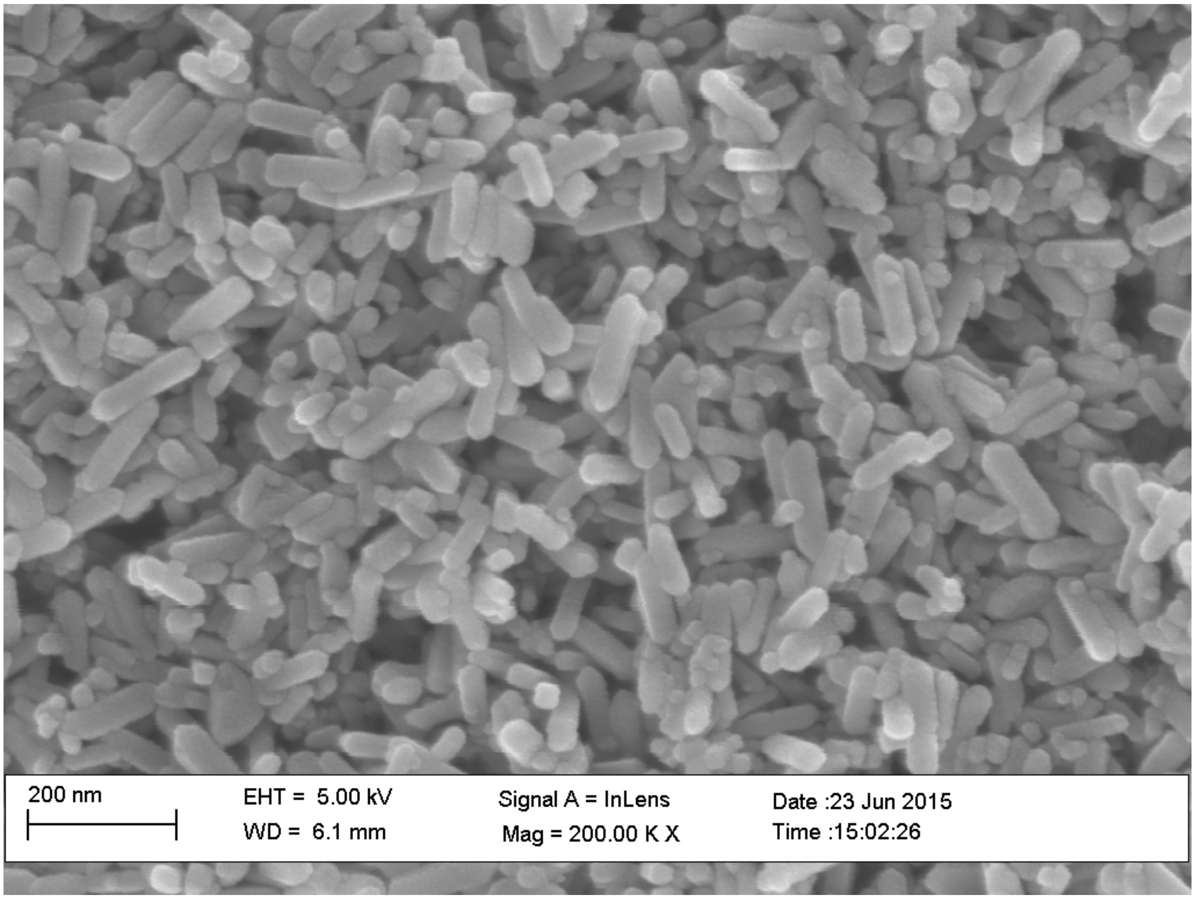
200 nm
EHT = 5.00 kV
WD = 6.1 mm
Signal A = InLens
Mag = 200.00 K X
Date :23 Jun 2015
Time :15:02:26

Brookite

S1b - HRTEM images of anatase, brookite and rutile films

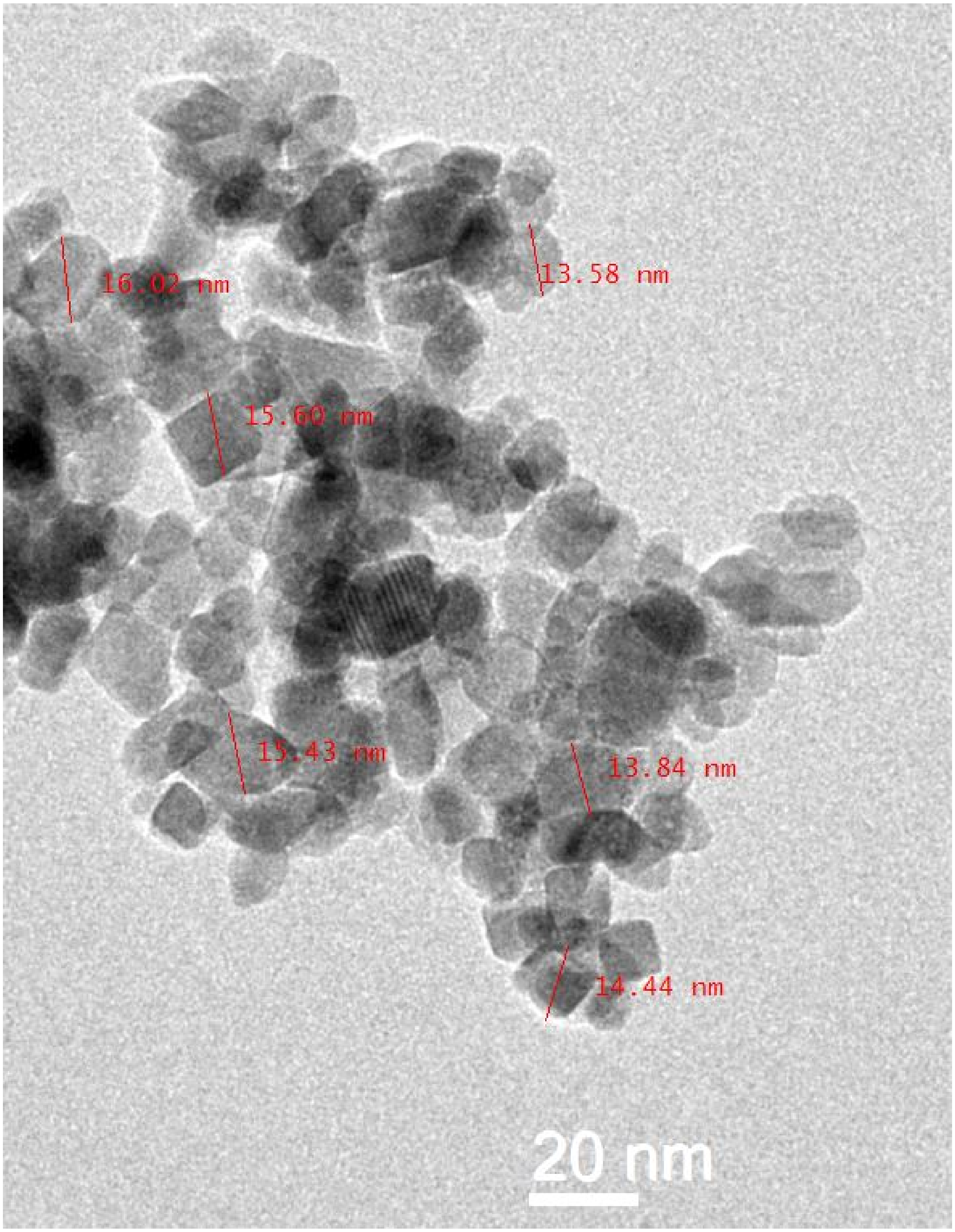

Anatase

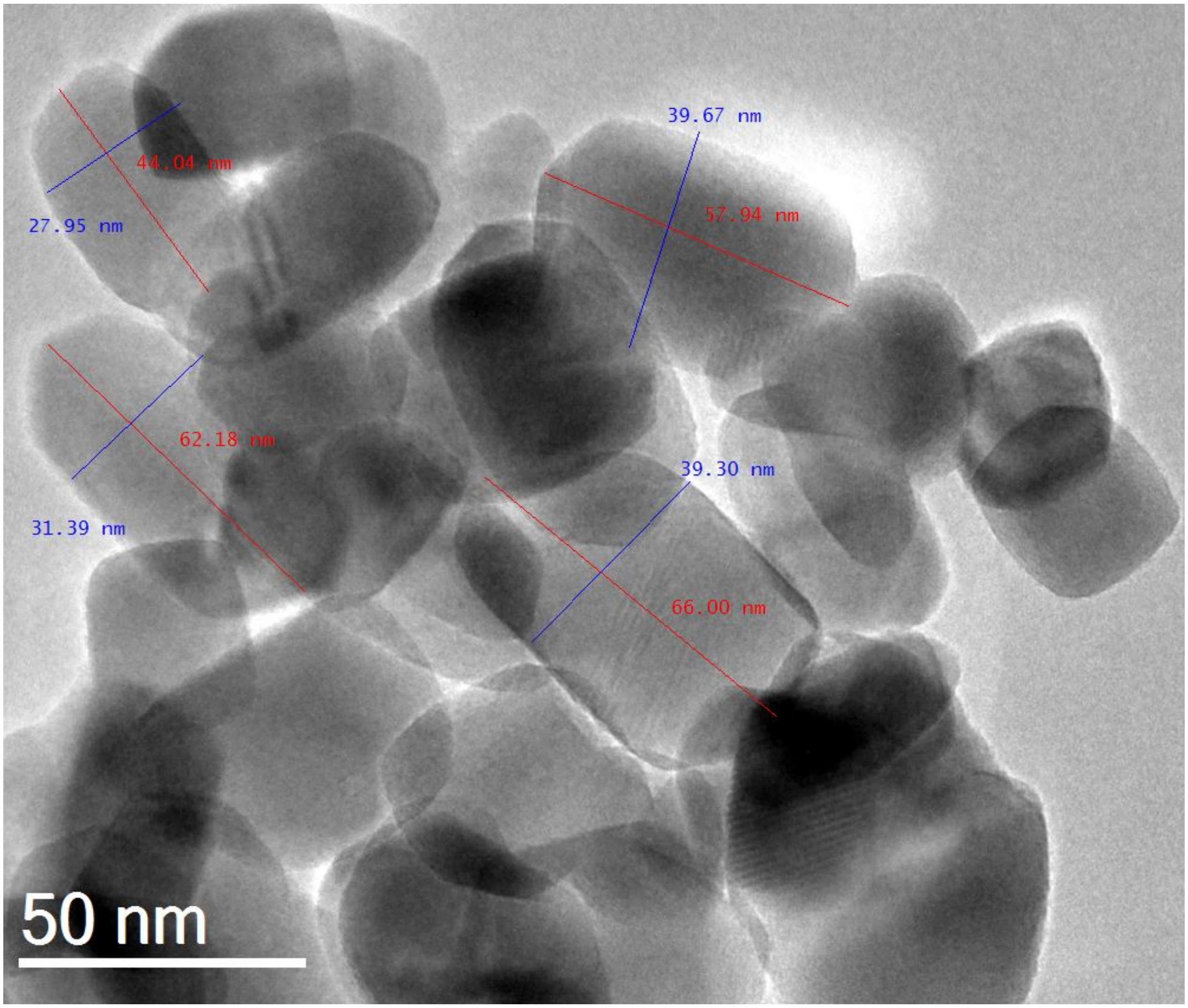
39.67 nm
44.04 nm
57.94 nm
27.95 nm
62.18 nm
39.30 nm
31.39 nm
66.00 nm
50 nm
Rutile

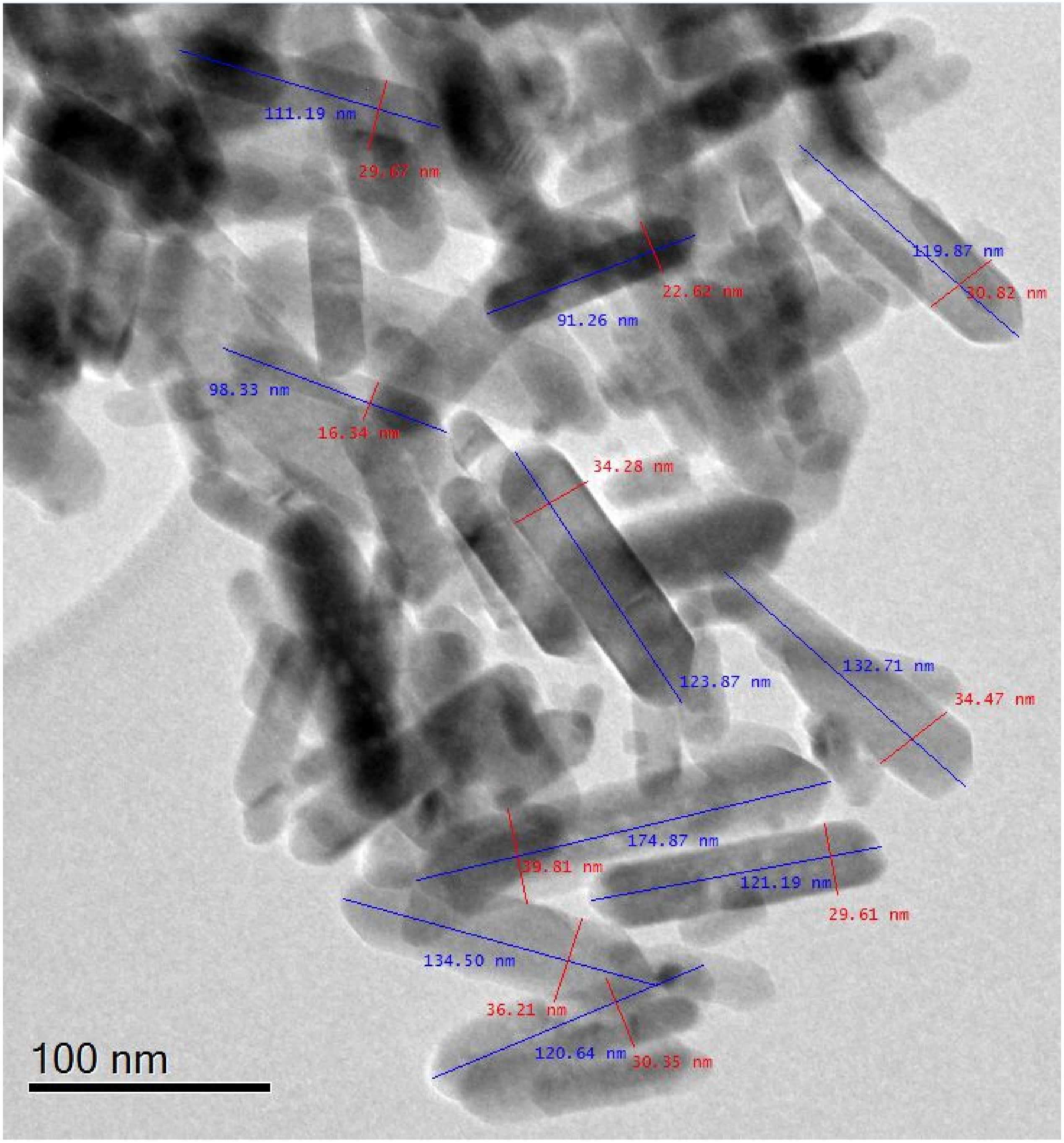
111.19 nm
29.67 nm
22.62 nm
91.26 nm
119.87 nm
30.82 nm
98.33 nm
16.34 nm
34.28 nm
123.87 nm
132.71 nm
34.47 nm
174.87 nm
39.81 nm
121.19 nm
29.61 nm
134.50 nm
36.21 nm
120.64 nm
30.35 nm
100 nm
Brookite

S2 - Results of LeBail refinement of PXRD data

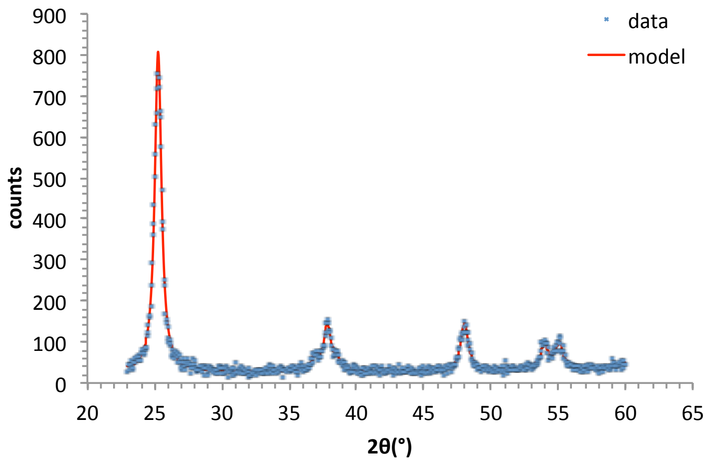

Anatase

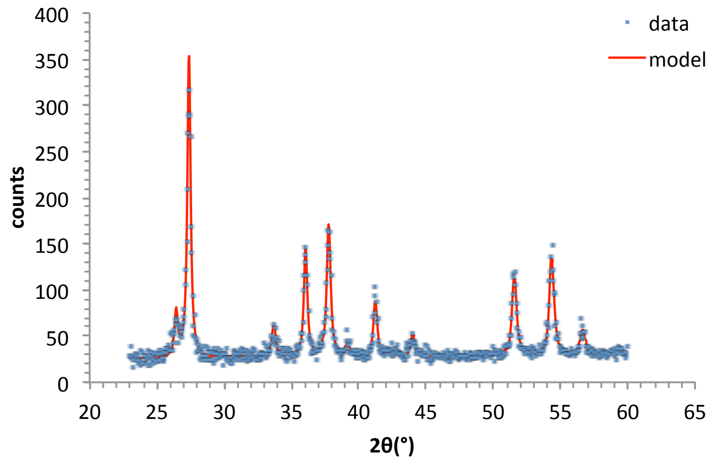

Rutile

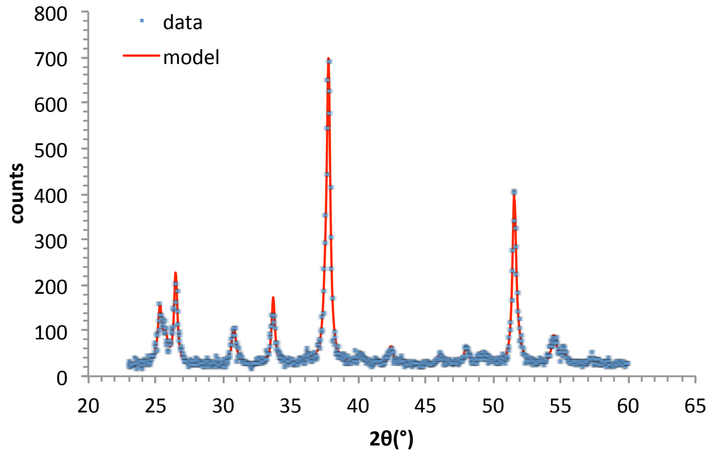

Brookite

S3 - Comparison of raman spectra of mesoporous anatase brookite and rutile to standard spectra

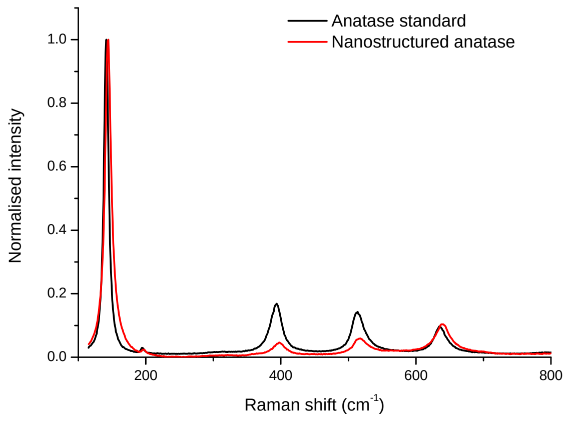

Anatase

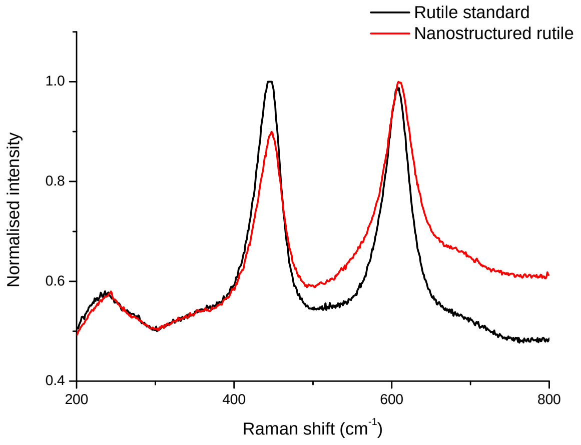

Rutile

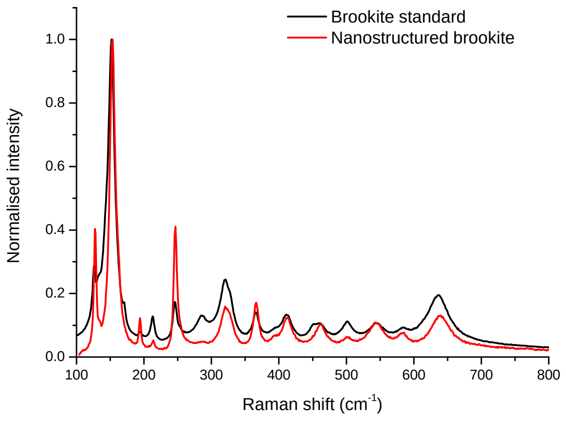

Brookite

S4 - Results of XPS with argon etching.

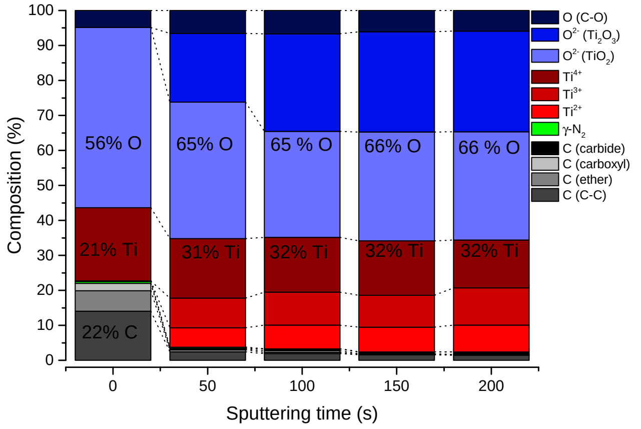

Anatase

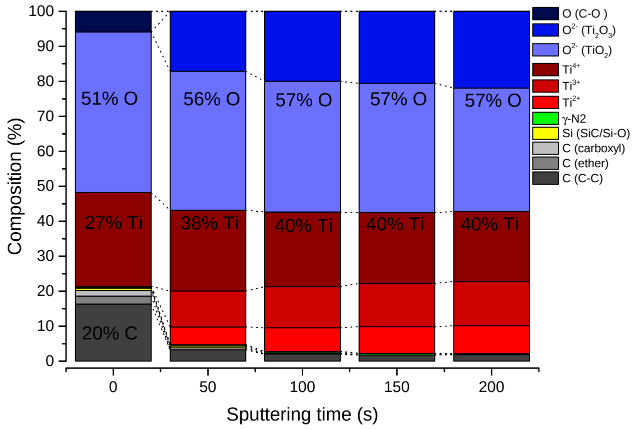

Rutile

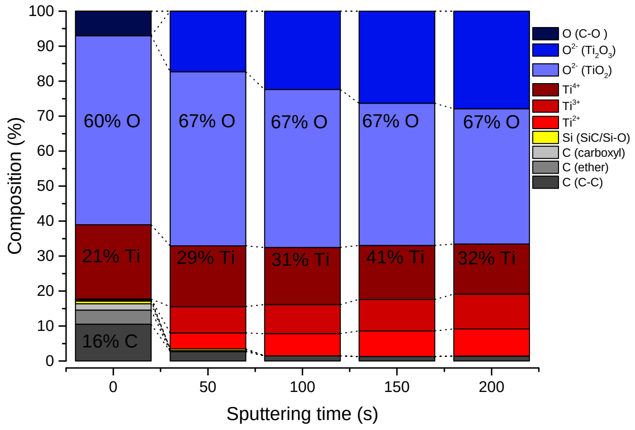

Brookite

S5 - (Indirect allowed) Tauc plots

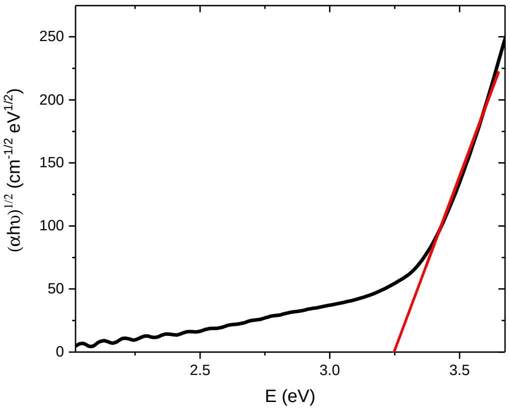

Anatase indirect band gap

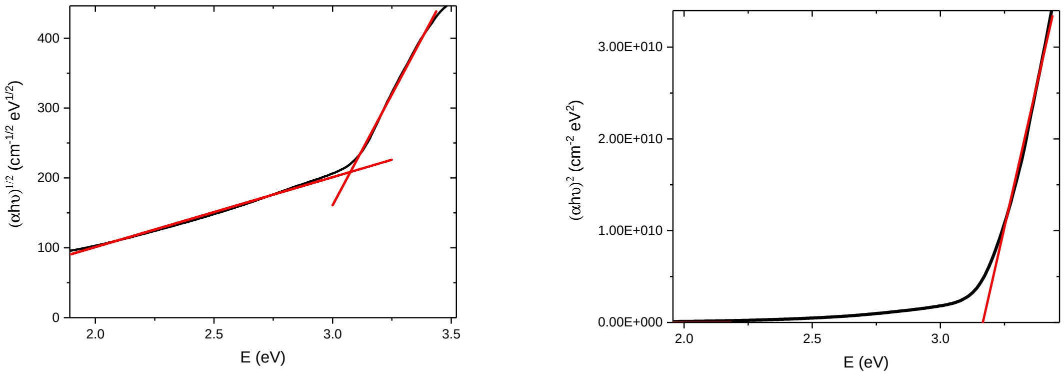

Rutile: indirect (left) and direct (right) band gaps

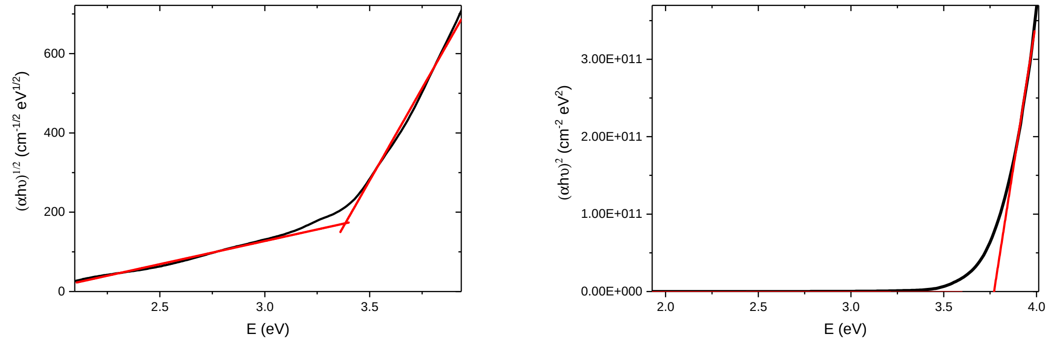

Brookite. Indirect (left) and direct (right) band gaps

Note that because of the higher degree of scattering in the mesoporous brookite and rutile films a more substantial ‘tail’ is observed before the indirect band gap. This is accounted for using a correction suggested by Chen and Miller [2], where the intercept of the absorption onset and the absorption tail is taken to be the optical band gap.

S6 - BET and BJH isotherms

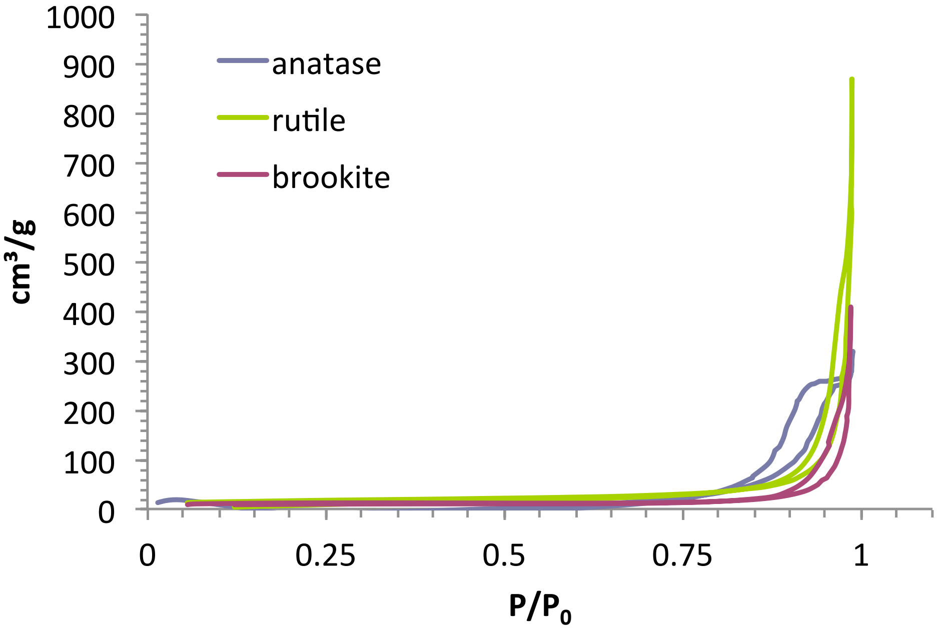

BET isotherm

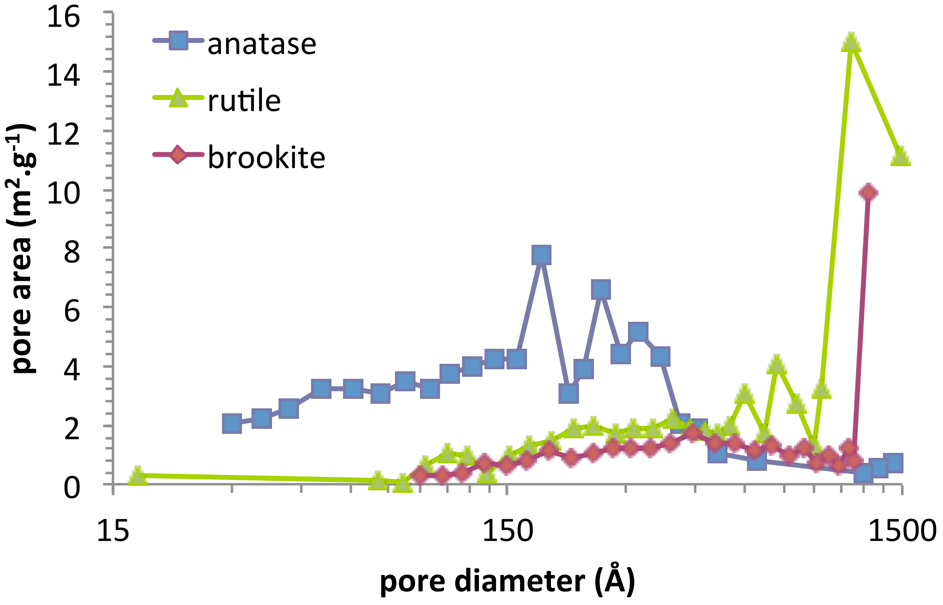

BJH isotherm

S7 - Absorptance and reflectance of mesoporous films

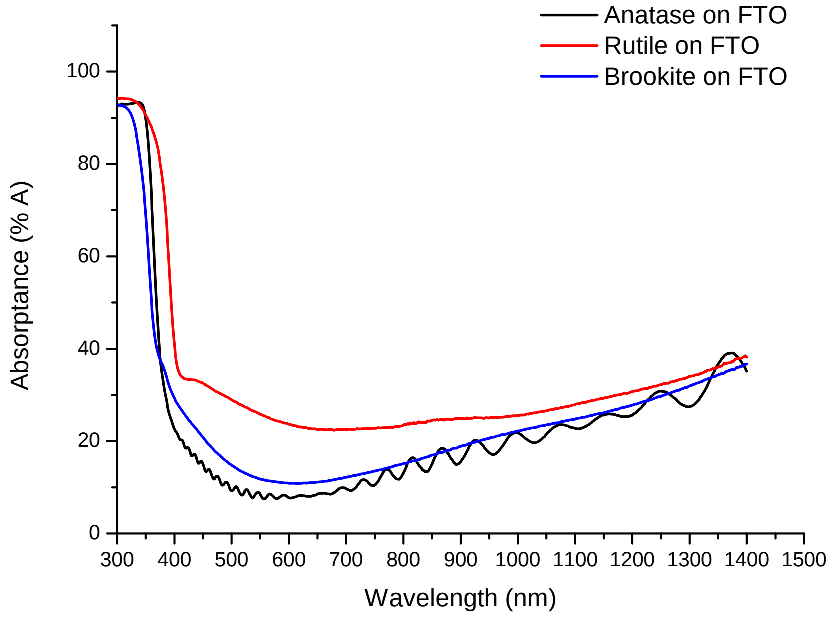

Absorptance

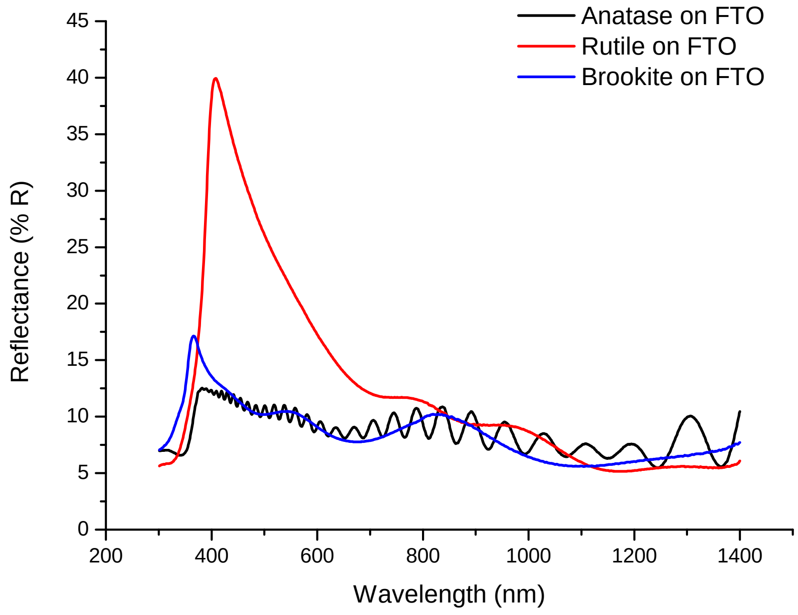

Reflectance

S8 - Transient spectra under argon

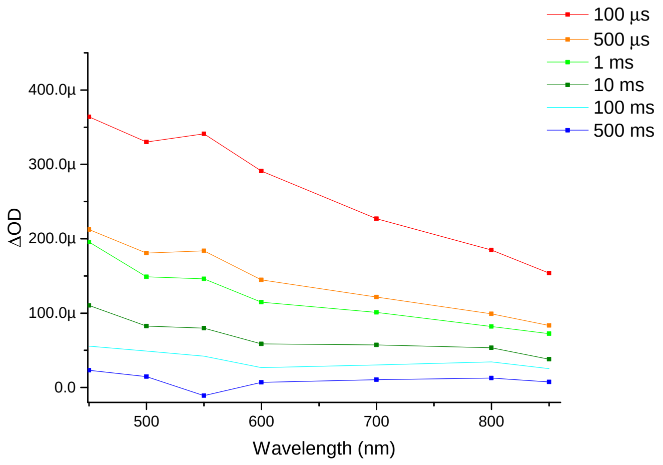

Anatase

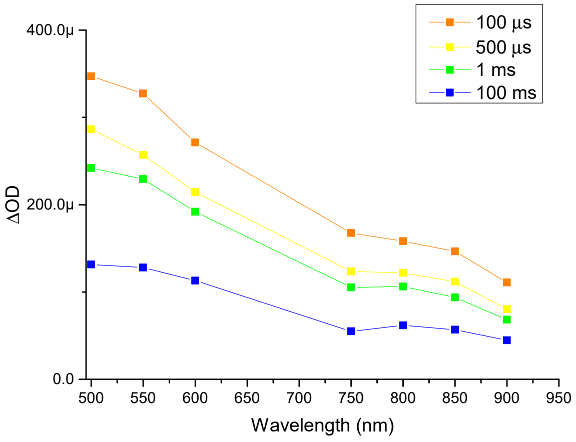

Rutile

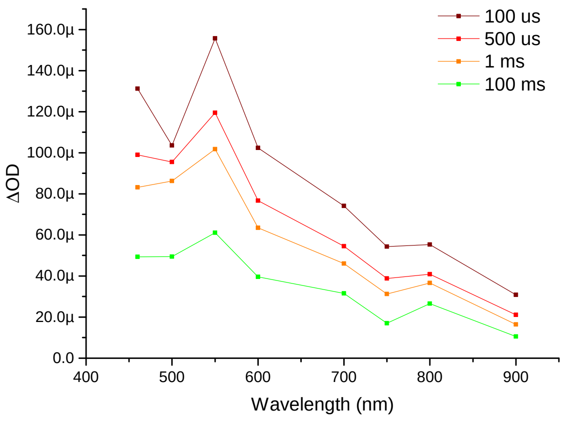

Brookite

S9 - Transient spectra in the presence of $AgNO_3$

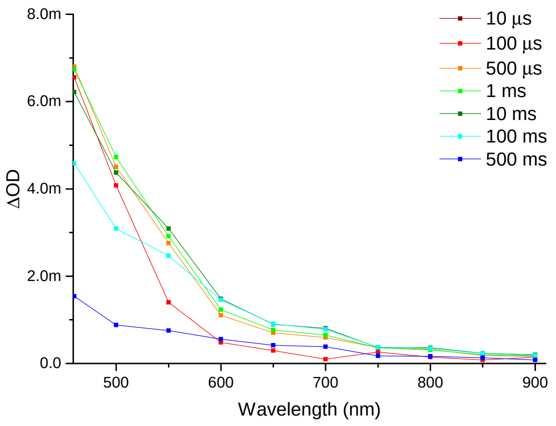

Anatase

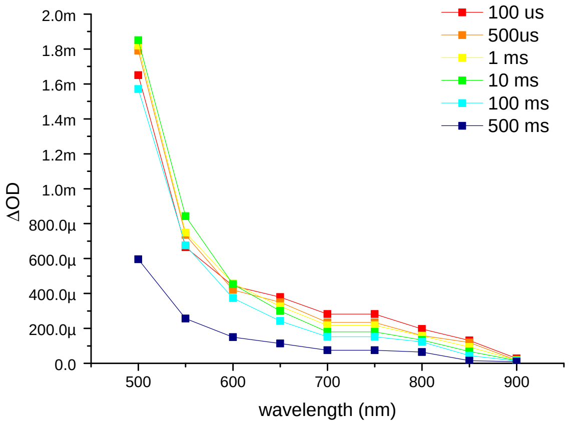

Rutile

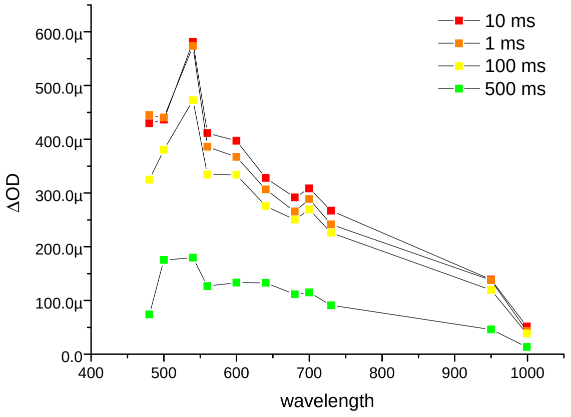

Brookite

S10 - Transient spectra in the presence of MeOH

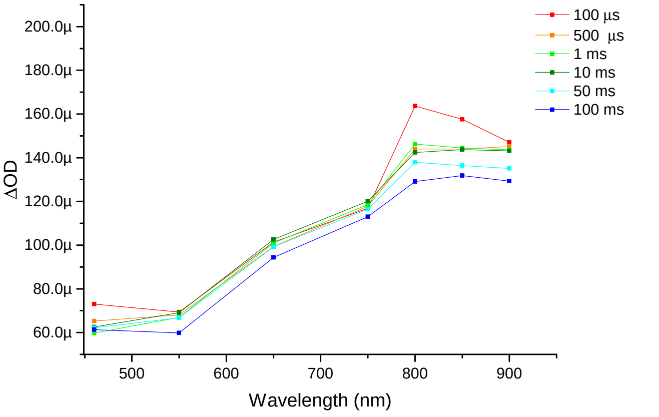

Anatase

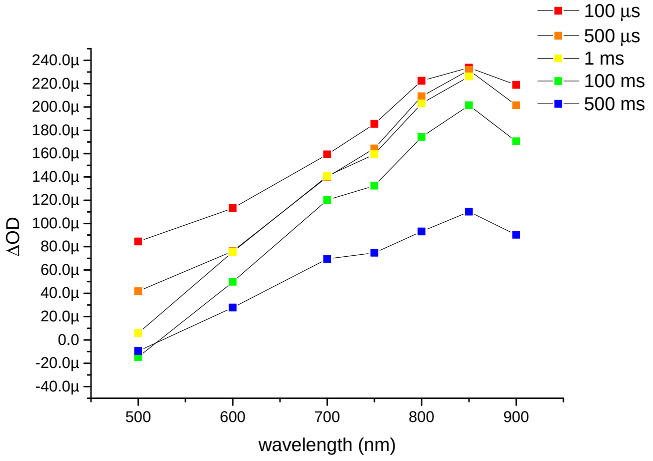

Rutile

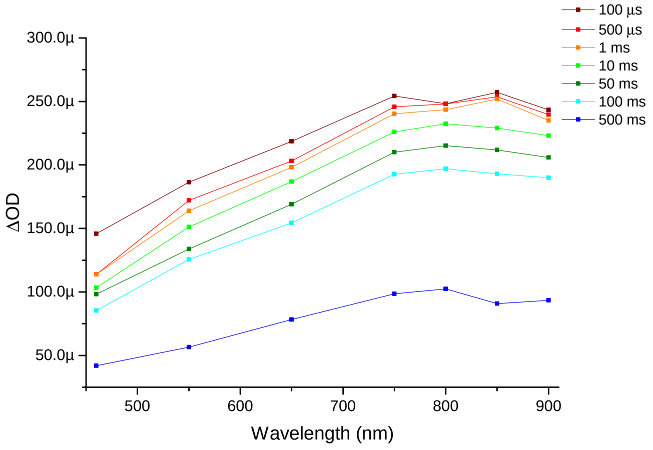

Brookite

S11 - Simulation of the recombination dynamics of rutile.

Details of the model and result.

To demonstrate a model in which both where the decay behavior of rutile may be reproduced via a combination of thermal walking and tunneling, we carry out a Monte Carlo simulation. In this simulation the electrons are given a probability to tunnel to another site or walk to an adjacent site depending on thermal excitations. We have based our model on that of J. Nelson *et al.* in [4].

Briefly, the current model is an amalgamation of two different models: the continuous time random walk (CTRW) and long range tunneling or 'hopping' model. In the CTRW a nearest neighbor site is chosen randomly and the time is advanced by $(\ln X)\frac{e^{a/a_B(E_F)}}{k_0}$, where X is a random number between 0 and 1, a is the lattice spacing, $a_B(E)$ is the effective bohr radius, assumed to take the form $a_B(E) = a_0/\sqrt{E_C - E}$ with a_0 a constant and E_C being the conduction band energy. In the hopping model the electrons can only move from site to site through a tunneling process, the probability of which is determined by the Miller-Abrahams model. A site is chosen at random with a probability of $\Pr(\text{site } i \rightarrow \text{ site } j) = k_{ij}/\sum_{l\neq i} k_{il}$ with k_{ij} given by

$$k_{iS} = k_S e^{-r_{iS}/a_B(E_i)} \tag{1}$$

for tunneling from site i to the hole, and

$$k_{ij} = k_0 e^{-r_{ij}/a_B(E_i+E_{ij})} e^{-E_i j/kT} \tag{2}$$

for the other sites. Here k_S and k_0 represents the strength of the electron-hole and electron-vacant site trap interaction respectively, while r_{iS}, r_{ij} represents the distance between the electron and hole, and the distance between the electron and site. E_i is the site energy, E_{ij} is the maximum of $E_j - E_i$ and 0. k is the usual Boltzmann constant. The time is then advanced by $(\ln X)/k_{iS}$ or $(\ln X)/k_{ij}$ depending on whether the destination site is a hole or a vacant site, and X is a random number between 0 and 1. [1] In both models the electrons move from site to site until a hole site is reached at which recombination occurs and the simulation ends. For a more thorough discussion of the CTRW see [3].

In the current work, an attempt is made to incorporate long range tunneling into the CTRW. We assume that in order to carry out the CTRW the electron needs to be excited into the conduction band where it then walks to the next site. If it cannot be excited into the conduction band then it will tunnel to a vacant site. This is achieved by allowing the electrons to tunnel if it cannot be thermally activated to the conduction band. The probability that it can be excited into the conduction band is given by a Boltzmann factor $e^{-(E_c-E)/kT}$, where E_c is the conduction band energy and E is the site energy of the occupied site, and represents the probability of being excited into the conduction band.

In contrast to the models in [4], our model assumes that the holes are distributed throughout the particle as opposed to being on the surface. As we have included long range tunneling effects, we have dropped the assumption that the dominant mechanism is through thermal excitation into a nearest neighbor without any long range interactions. In addition to this, for our particular discussion we have also assumed that there are equal numbers of each of electrons and holes since studies of dye sensitized solar cells has shown log-linear behavior at the limit where electron concentration tends towards that of holes [4] (this is reflected in the fraction of holes (ϕ), which is fixed such that there is one hole is per lattice). A further assumption that has been relaxed is that the k_S will only be slightly larger than k_0. Unlike models for dye sensitized titania [4], where an electron must recombine with a cation coordinated to a surface bound molecule, holes in our model are found throughout the particle. As the most efficent and comonly used dyes minimise interaction between the molecule and the semiconductor [5], we hypothesized that modelling recombination with holes inside a rutile particle would require a stronger electron/hole interaction (i.e. larger value of k_s). This was borne out in the model, as more log-linear decays begin to appear when k_s is three to five orders of magnitude greater than k_0 (c.f. a two order of magnitude difference that is used to model recombination dye sensitized titania. [4])

A $15 \times 15 \times 15$ cubic lattice was with a lattice spacing of 4Å. Following [4], we use a value of $a_0 = 4a$. The vacant traps are given an exponential energy distribution from the conduction band. The band gap used in these simulations is 3.03 eV and an approximate electron quasi Fermi level for traps at 1.79 eV above the valence band, according to [7] (as will be discussed, the model is relatively insensitive to reasonable changes in this parameter). The simulation was run a thousand times for a hundred randomly generated energy configurations (n) each with the same parameters (discussed later). It was found that for one electron and one hole, only the variations of the vacant site trap depths and the ratio k_S/k_0 affected the decay behavior in any significant way. Furthermore for very shallow trap depths, the resulting decay very closely resembles those of the CTRW withour hopping. The trap depths were mainly responsible

for the shape of the decay whist the interaction strength ratio mainly affects the timescale. By varying these two variables a fitting to the raw data was made possible at a mean trap depth of 1.5 eV and $k_S/k0 = 10^5$. Note that these simulations are *not exhaustive* and more work is needed for a complete understanding of these simulations.

The vacant site energy distribution for electrons (which we use to approximate the electron DOTS) is well known to be an exponential tail in titanium dioxide. [3, 4] Thus a simple exponential decay function was used for the vacant site distribution. The mean energy of the distribution is the inverse of α/kT, a parameter representing the steepness of the experiential decay. During the simulation the lattice sites were randomly assigned an energy from the exponential decay whilst the hole energy was normally distributed. To sample the trap distribution, the lattice was re-initialized in a new energy configuration after a statistically significant number (m) of recombination events ('runs') was recorded. This allows averaging of the distribution of hole energy by collecting n configurations of m runs. The parameters used in the figure below are summarized in the following table.

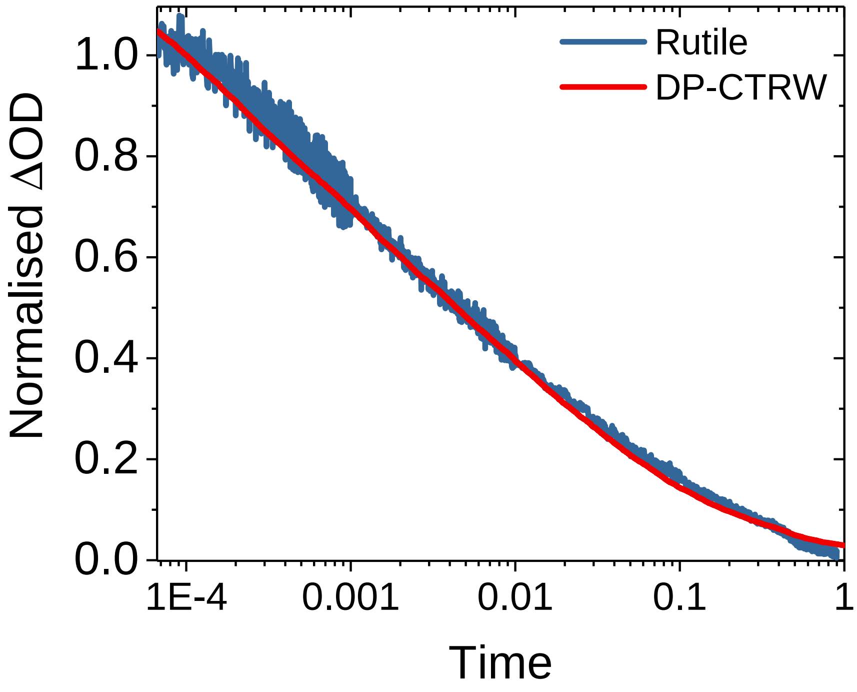

S11a Optimized result of the dual process continuous time random walk (DP-CTRW) recombination model (red), which combines a CTRW with a hopping model, compared to normalized TA decay of rutile (blue).

Lattice size	n	m	ϕ	α/kT (eV^{-1})	a (Å)	a_0	k_s (s^{-1})	k_0 (s^{-1})	T (K)
$15 \times 15 \times 15$	1000	100	2.96×10^{-4}	0.6667	4	a	2×10^9	1.5×10^4	300

Discussion of results and sensitivity analysis

In the pure walking (CTRW) model, recombination takes place across a wide range of time scales as expected for a diffusion process. The pure hopping and dual process hopping and walking model (DP-CTRW) on the other hand exhibit a two phased recombination process. Here, a fast recombination phase is followed by a slower, largely log-linear recombination phase. This is represented by the steep decline at earlier times, a stable region and then a relatively flat decay tail at later times. This is summarized in *Figure S10b*, which compares the recombination dynamics of the CTRW, optimized DP-CTRW, and pure hopping model.

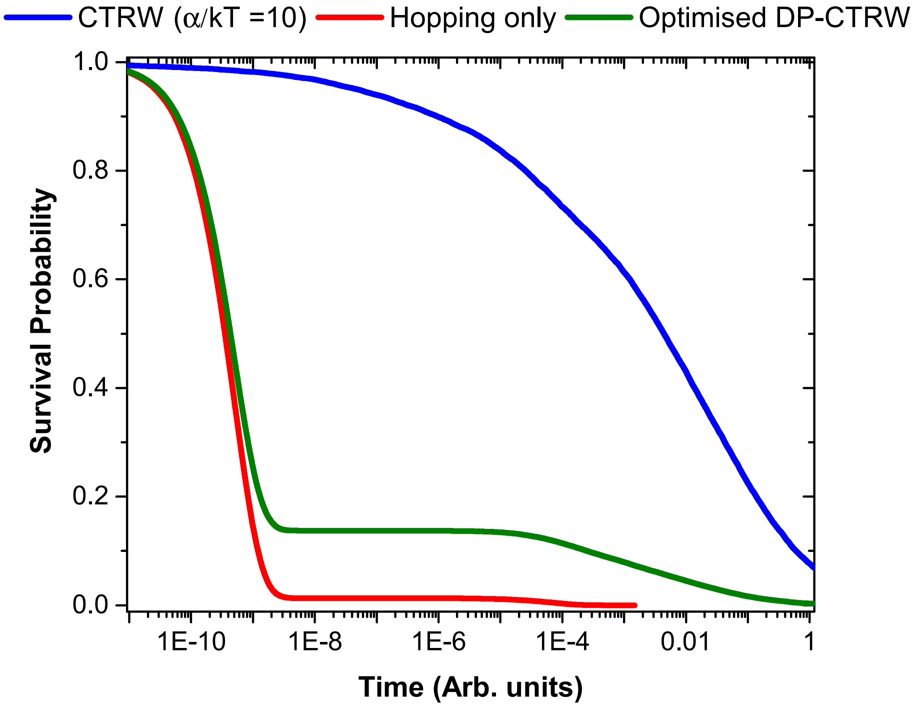

S11b Recombination dynamics for 'hopping' (tunneling) recombination, a continuous time random walk (CTRW) and a combined, dual process continuous time random walk (DP-CTRW). The parameters used in the DP-CTRW and pure hopping model are the same, and are enumerated on the previous page.

The optimization process showed that only changes to k_s, k_0 and $\frac{\alpha}{kT}$ and induce significant changes to the recombination dynamics. The influence of these parameters on recombination dynamics will be briefly discussed. As can be seen in Figure *S10c*, a decrease in the electron-hole affinity k_S results in a delayed rapid phase. This behavior is due to two factors - an increased waiting time for tunneling and a decreased probability for tunneling. Similarly from Figure *S10c*, the effect of an increase in α is that vacant traps that are are shallower, i.e. closer to the conduction band. This gives an increased waiting time for walking and an increased probability of walking (CTRW).

For the optimized DP-CTRW model, the differences to the pure CTRW and pure hopping models can be explained by the factors above. The large electron hole affinity and a small α used to fit the data produce a the rapid phase which approximates ultra fast TA recombination dynamics in rutile to within an order of magnitude [6]. Further, our analysis shows this initial recombination is dominated by tunneling due to the high electron-hole affinity. However, due to the walking (CTRW) mode, a small but significant fraction of walkers survive, which later recombine in the log-linear regime. The presence of the CTRW mode in the DP-CTRW however, means that there is a small but significant probability that the electron would walk instead of tunnel, especially those caught in shallow traps. These 'slow' walkers are responsible for the log-linear decay. However, our analysis shows that even at late times, the recombination step is still almost exclusively a tunneling step. Further, the presence of a large proportion of fast decays and by counting the number of walks and hops demonstrates that overall, the model is *tunneling dominated.* We attribute presence of the stronger log-linear phase to be mostly a result of a smaller proportions of straggling electrons that spend some time *walking before tunneling to the hole.* As illustrated in *Figure S10d*, which compares late dynamics of the three models, this behavior is unique to the DP-CTRW and is not present in either the CTRW or hopping models. That the simulation requires deeply trapped electrons and immobile holes results in a tunneling

dominated regime to closely fit the data supports the hypothesis that electrons and holes are deeply trapped and must consequently predominantly recombine through tunneling, which at late times is mediated by a small fraction of high energy electron walkers that slows the recombination process in the slow phase. Deep trapping, combined with a small fraction of thermally activated walkers results in the optimized DP-CTRW being temperature independent over a temperature range in which water spitting could plausibly occur. This is demonstrated in *Figure S10e*.

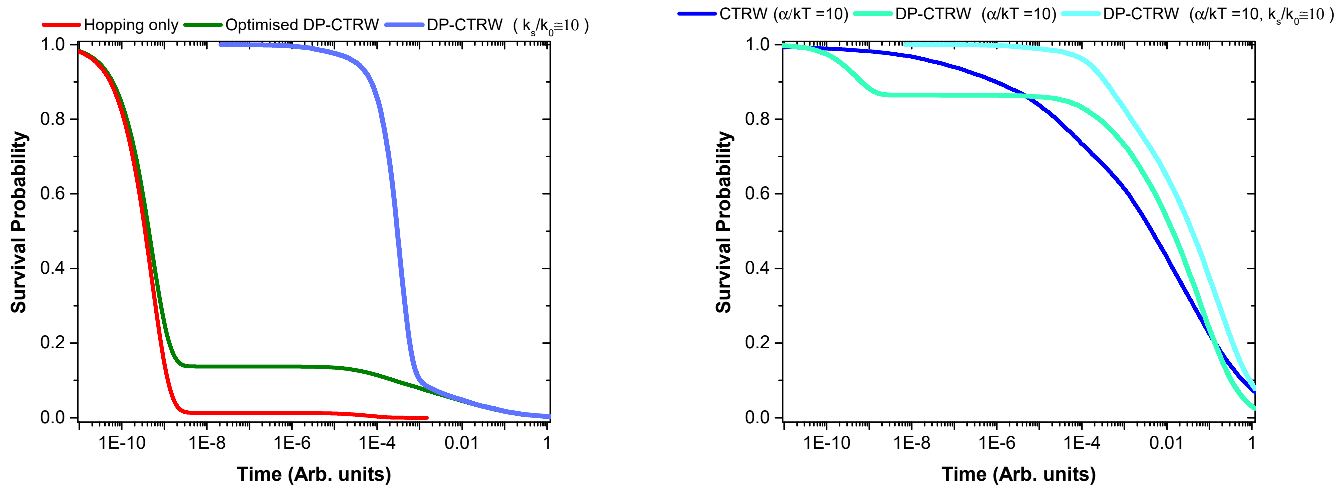

S11c Decreasing hole affinity (k_s) WRT lattice affinity (k_0) slows the rapid tunneling phase (left). Increasing α results in recombination dominated by walking (right)

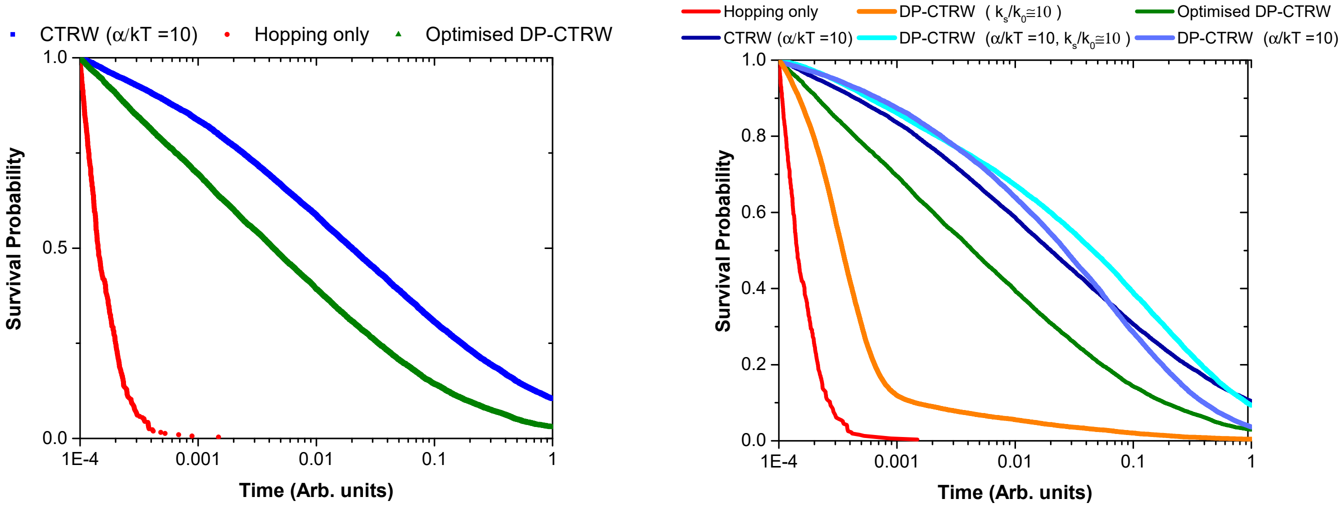

S11d Comparison of the recombination dynamics of the optimised DP-CTRW to the pure tunneling and walking (CTRW) models on at late times (left) normalized to 100 μs. The optimized DP-CTRW 'linearizes' as its electron traps are deep *and* it has strong tunneling recombination. This is seen by comparing un-optimized DP-CTRW to the pure models (right). Increasing α alone results in late dynamics similar to walking. Decreasing k_s results retards the early recombination phase, producing bi-phasic late dynamics with an early phase similar to pure hopping and a small log linear 'tail'. In the extreme case where α is large and k_s is small with respect to k_0, it appears that α is the dominant factor as walking appears to dominate the late dynamics.

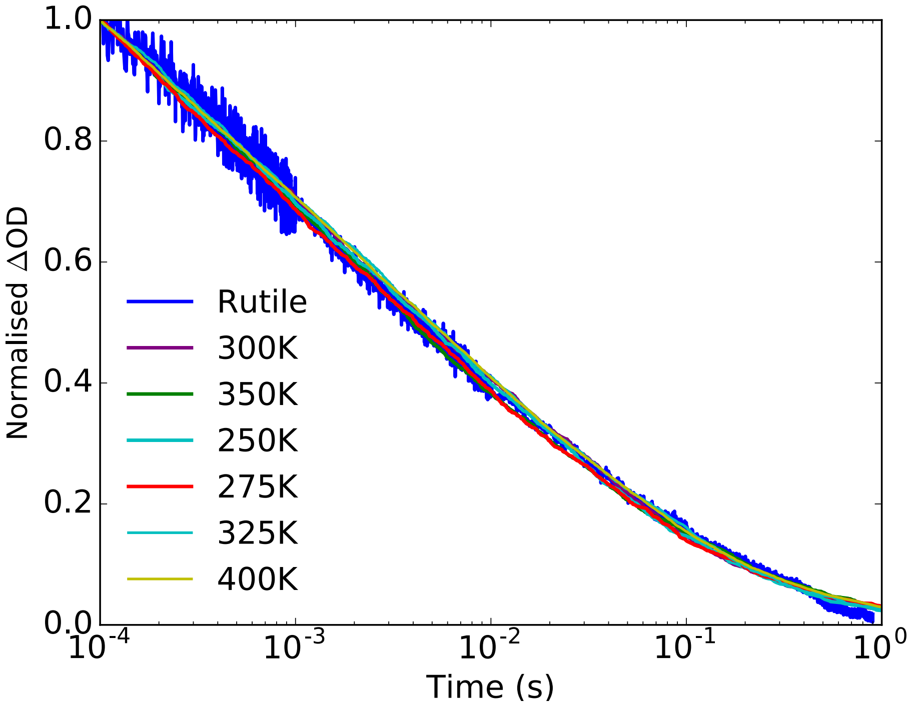

S11e The optimized DP-CTRW shows no significant temperature dependence over the temperature range in which water splitting could plausibly occur. For the purposes of comparison, DP-CTRW decays at different temperatures are shown alongside the TA decay of rutile.

S12 - Excitation intensity dependence on the charge carrier dynamics mesoporous anatase, brookite and rutile films

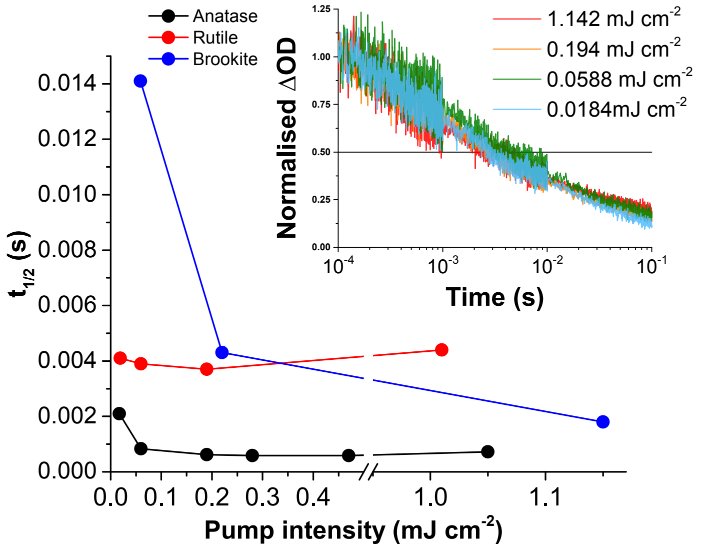

S12a Intensity dependence on half time of mesoporous anatase, brookite and rutile films

The negligible change in the half time of rutile (see inset) indicates a pseudo first order regime. This is consistent with the high donor density of rutile. The results of simulation remain consistent with this result as simulation produces a sum of stretched exponential type decays - decay forms which are related to dispersive pseudo first order

recombination [3, 4]. In contrast, anatase and brookite exhibit a large increase in half time (compared to error) at low intensities. This indicates the beginning of a transition from second order to pseudo first order recombination. Because of the (relativity) strong n-type character of brookite and anatase, this effect is comparatively minor. As a result, at all intensities measured, the decay dynamics of the three phases did not significantly change with decreasing intensity, with anatase and brookite exhibiting power law decays and rutile exhibiting a log decay. This is shown in *Figure S12b*

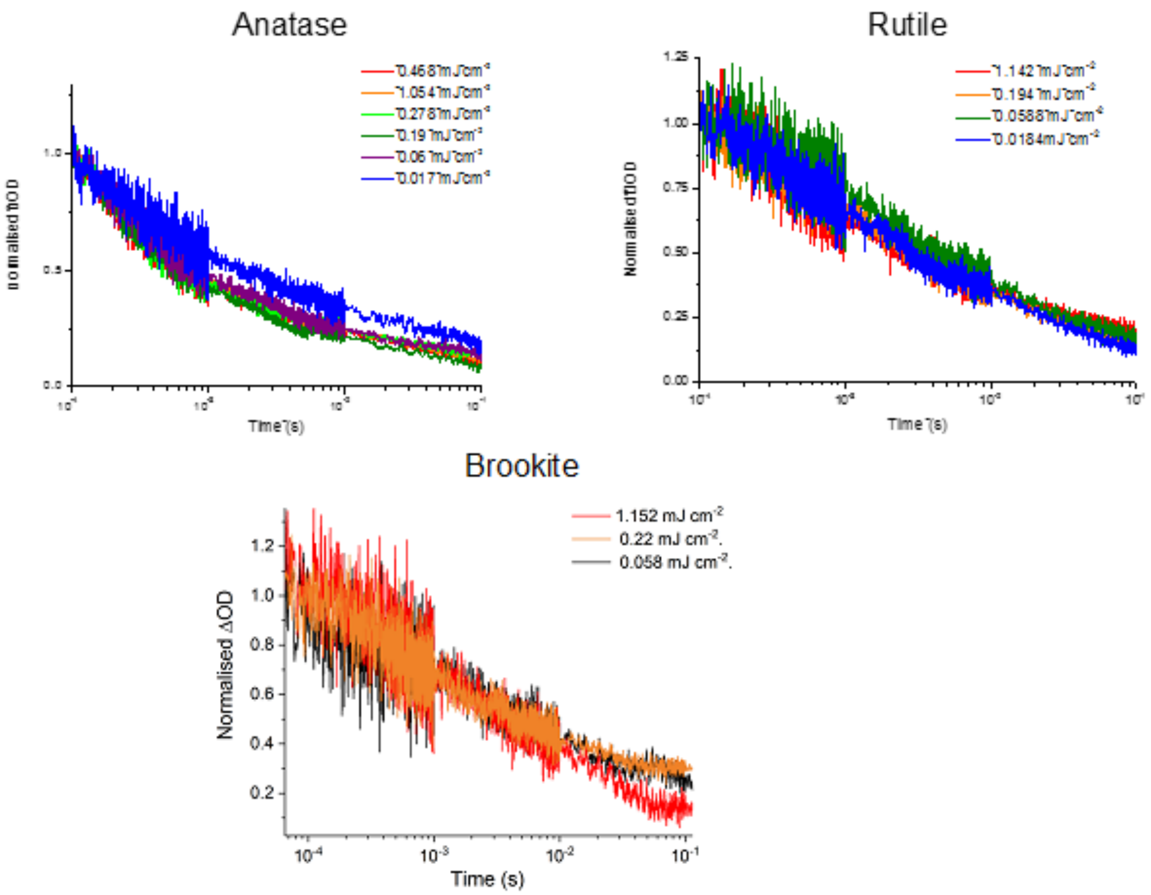

S12b Intensity dependence on the decay dynamics of mesoporous anatase, brookite and rutile films

References

[1] H Bässler. Charge Transport in Disordered Organic Photoconductors a Monte Carlo Simulation Study. *physica status solidi (b)*, 175(1):15–56, 1993.

[2] Z Chen, H Dinh, and E Miller. *Photoelectrochemical Water Splitting: Standards, Experimental Methods, and Protocols*. SpringerBriefs in Energy. Springer New York ; Imprint: Springer, 2013.

[3] Jenny Nelson and Rosemary E Chandler. Random walk models of charge transfer and transport in dye sensitized systems. *Coordination Chemistry Reviews*, 248(13-14):1181–1194, 2004.

[4] Jenny Nelson, Saif A Haque, David R Klug, and James R Durrant. Trap-limited recombination in dye-sensitized nanocrystalline metal oxide electrodes. *Phys. Rev. B*, 63(20):205321, May 2001.

[5] Anna Reynal, Amparo Forneli, and Emilio Palomares. Dye structure–charge transfer process relationship in efficient ruthenium-dye based dye sensitized solar cells. *Energy Environ. Sci.*, 3(6):805–812, 2010.

[6] Michael Sachs, Ernest Pastor, Andreas Kafizas, and James R Durrant. Evaluation of Surface State Mediated Charge Recombination in Anatase and Rutile TiO2. *The Journal of Physical Chemistry Letters*, 7(19):3742–3746, 2016.

[7] Ming Zhu, Yang Mi, Gangbei Zhu, Deyong Li, Yunpeng Wang, and Yuxiang Weng. Determination of Midgap State Energy Levels of an Anatase TiO 2Nanocrystal Film by Nanosecond Transient Infrared Absorption – Excitation Energy Scanning Spectra. *Journal of Physical Chemistry C*, 117(37):18863–18869, September 2013.
